# TET2 regulates immune tolerance in chronically activated mast cells

**DOI:** 10.1172/jci.insight.154191

**Published:** 2022-04-08

**Authors:** Riccardo Rigo, Rabie Chelbi, Julie Agopian, Sebastien Letard, Aurélien Griffon, Hussein Ghamlouch, Julien Vernerey, Vasileios Ladopoulos, Edwige Voisset, Paulo De Sepulveda, Geoffrey Guittard, Jacques A. Nunès, Ghislain Bidaut, Berthold Göttgens, Michael Weber, Olivier A. Bernard, Patrice Dubreuil, Erinn Soucie

**Affiliations:** 1 Cancer Research Center of Marseille (CRCM), INSERM, CNRS, Aix-Marseille University, Institut Paoli-Calmettes, Equipe Labélisée Ligue Nationale Contre le Cancer, Marseille, France.; 2 Inovarion, Paris, France.; 3 INSERM, Mixed Research Unit (UMR) 1170, Institut Gustave Roussy, Facility of Medicine, Paris-Sud University, Paris-Saclay University, Equipe Labélisée Ligue Nationale Contre le Cancer, Villejuif, France.; 4 CRCM, Institut Paoli-Calmettes, Inserm, CNRS, Aix Marseille University, Marseille, France.; 5 Department of Haematology, Cambridge Institute for Medical Research, and; 6 Wellcome-MRC Cambridge Stem Cell Institute, University of Cambridge, Cambridge, United Kingdom.; 7 CNRS, University of Strasbourg, UMR7242 Biotechnology and Cell Signaling, Illkirch, France.

**Keywords:** Inflammation, Epigenetics

## Abstract

Mutation of the TET2 DNA-hydroxymethylase has been associated with a number of immune pathologies. The disparity in phenotype and clinical presentation among these pathologies leads to questions regarding the role of TET2 mutation in promoting disease evolution in different immune cell types. Here we show that, in primary mast cells, *Tet2* expression is induced in response to chronic and acute activation signals. In TET2-deficient mast cells, chronic activation via the oncogenic *KITD816V* allele associated with mastocytosis, selects for a specific epigenetic signature characterized by hypermethylated DNA regions (HMR) at immune response genes. H3K27ac and transcription factor binding is consistent with priming or more open chromatin at both HMR and non-HMR in proximity to immune genes in these cells, and this signature coincides with increased pathological inflammation signals. HMR are also associated with a subset of immune genes that are direct targets of TET2 and repressed in TET2-deficient cells. Repression of these genes results in immune tolerance to acute stimulation that can be rescued with vitamin C treatment or reiterated with a Tet inhibitor. Overall, our data support a model where TET2 plays a direct role in preventing immune tolerance in chronically activated mast cells, supporting TET2 as a viable target to reprogram the innate immune response for innovative therapies.

## Graphical abstract

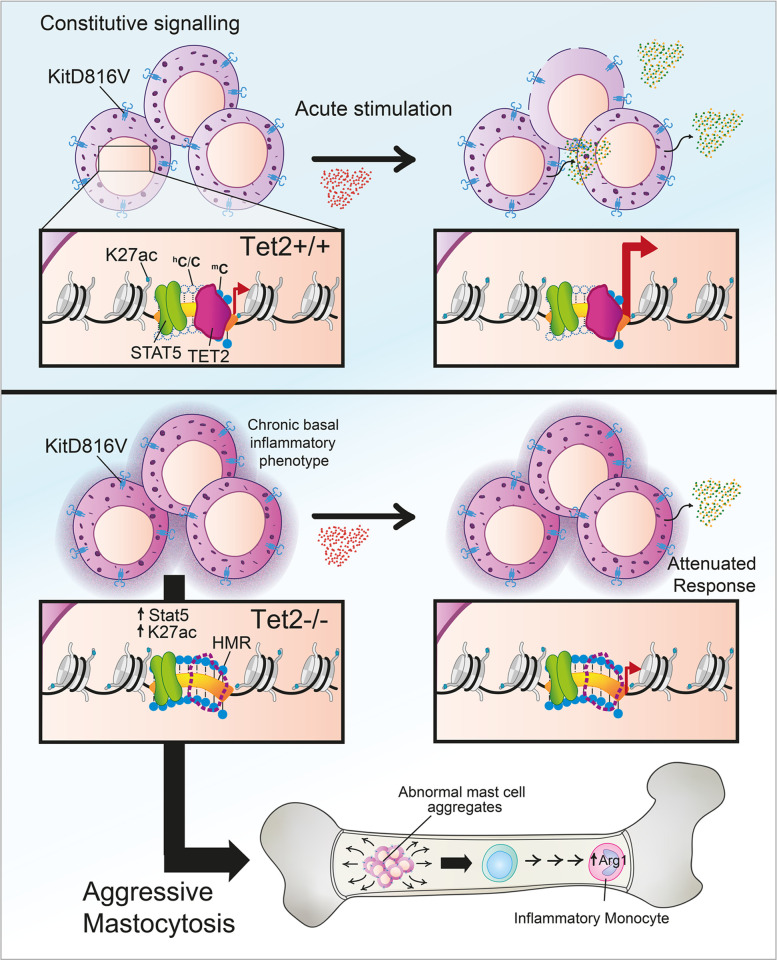

## Introduction

Ten-eleven translocation-2 (TET2) is an epi-regulator that belongs to the family of α-ketoglutarate–dependent hydroxylases that catalyses essentially the oxidization of 5-methylcytosine (5mC) into 5-hydroxymethylcytosines (5hmC), which is a step toward cytosine demethylation (1). TET2 loss-of-function mutations have been widely reported in human myeloid and lymphoid malignancies (1, 2). However, TET2 mutation alone is not sufficient to drive malignant disease (3–5). Information concerning the molecular role of TET2 mutation in cooperation with “driver” mutations has largely been garnered from genetic mouse models of acute myeloid leukemia (AML), including *TET2*^–/–^ *Flt3-ITD* and *TET2*^–/–^ *AML-ETO* mice (6, 7). In these models, TET2 loss of function is associated with DNA hypermethylation at regulatory regions of lineage and differentiation genes whose deregulation in a TET2-deficient context is consistent with the transformed phenotype of these leukemia. TET2 loss-of-function mutations also correlate with aggressive forms of mastocytosis, a rare immune disease in humans (8–10). The frequency of TET2 mutation in patients with mastocytosis is 20%–30%; however, < 1% of patients with mastocytosis develop mast cell leukemia (8, 10–12). As such, it’s unclear to what extent TET2’s role in leukemia applies in the context of this immune disorder.

More recent studies show that *TET*2 expression is transiently induced in response to acute activation signals in innate immune cells (13–17). Upon induction, TET2 dynamically regulates gene expression and activation of the inflammasome downstream of inflammation signals in macrophages (13–15), and myeloid-specific deletion of *TET2* changed the immunosuppressive transcriptomic signature of TAMs to promote an inflammatory gene signature in a murine model of melanoma (16). Taken together, we wondered if TET2 mutation and cooperation with activating KIT mutations in mastocytosis could be an ideal model to further study the importance of *TET2* expression for innate immune cell function in differentiated immune cells.

As part of the innate immune system, mast cells generally reside in peripheral and connective tissues of the body. Mastocytosis is characterized by abnormal tissue invasion, accumulation, and immune activation of mast cells that ultimately impinge on organ function (12). Patients with aggressive forms of mastocytosis can also develop additional myeloid malignancies, termed associated hematological neoplams (AHN). These secondary diseases sometimes share a clone of origin with mastocytosis, detected by common mutational profiles, but they can also evolve in parallel (12, 18). In the majority of cases (>90%), the clonal proliferation of mast cells associated with adult disease is secondary to a gain-of-function mutation of the tyrosine kinase receptor KIT (KITD816V) (19). This mutation results in constitutive signaling through this immune receptor in the absence of its cognate ligand, stem cell factor (SCF) (12, 19–21). In addition to regulating genes involved in proliferation and survival, SCF signaling via the endogenous KIT receptor can enhance acute mast cell activation, although prolonged exposure to SCF can also result in attenuation of activation in mast cell cultures (20, 22). Together, expression of the *KITD816V* allele promotes mast cell survival and proliferation; however, the role of KITD816V in mast cell activation is less clear (22–25).

Both TET2 and KIT mutation are required to provoke a mastocytosis-like disease in mice (26), although hypermethylated DNA signatures, consistent with TET2 loss of function, have only been described in TET2-deficient mast cells (27). By combining genome-wide approaches, we now show that a distinct epigenetic landscape is selected for in TET2-deficient mast cells upon stable expression of the *KITD816V* allele. Our functional data in primary mast cells further support a model in which TET2 is required to fully induce the expression of a subset of immune genes downstream of KITD816V and other activation signals important to resolve chronic inflammation and prevent immune tolerance. Together, this provides a molecular explanation for the cooperation between TET2 and KITD816V mutations in driving aggressive forms of mastocytosis and adds to a growing body of evidence supporting an immune function for TET2 in differentiated hematopoietic cells.

## Results

### KITD816V selects for hypermethylated regions associated to immune function in TET2-deficient mast cells.

Primary murine mast cell cultures are stable and can be derived in vitro from BM of WT (*TET2*^+/+^) and TET2-deficient (*TET2*^–/–^) mice. Infection of these differentiated cells with lenti-virus (IRES-GFP) coding for the KITD816V allele reiterates the TET2 and KIT mutations found in patient mast cell clones. We have previously used this system to show a clonal advantage of *Tet2*^–/–^*KITD816V* over *Tet2*^+/+^*KITD816V* mast cells in vitro, similar to disease (8). Levels of KIT (endogenous and KITD816V) are not statistically different in infected compared with uninfected cells (GFP^+^ versus GFP^–^), and mast cell lineage markers including FCER1A and several mast cell–specific proteases are also similar in *Tet2^+/+^KITD816V* and *Tet2^–/–^KITD816V* cells (8).

**Figure 1:**
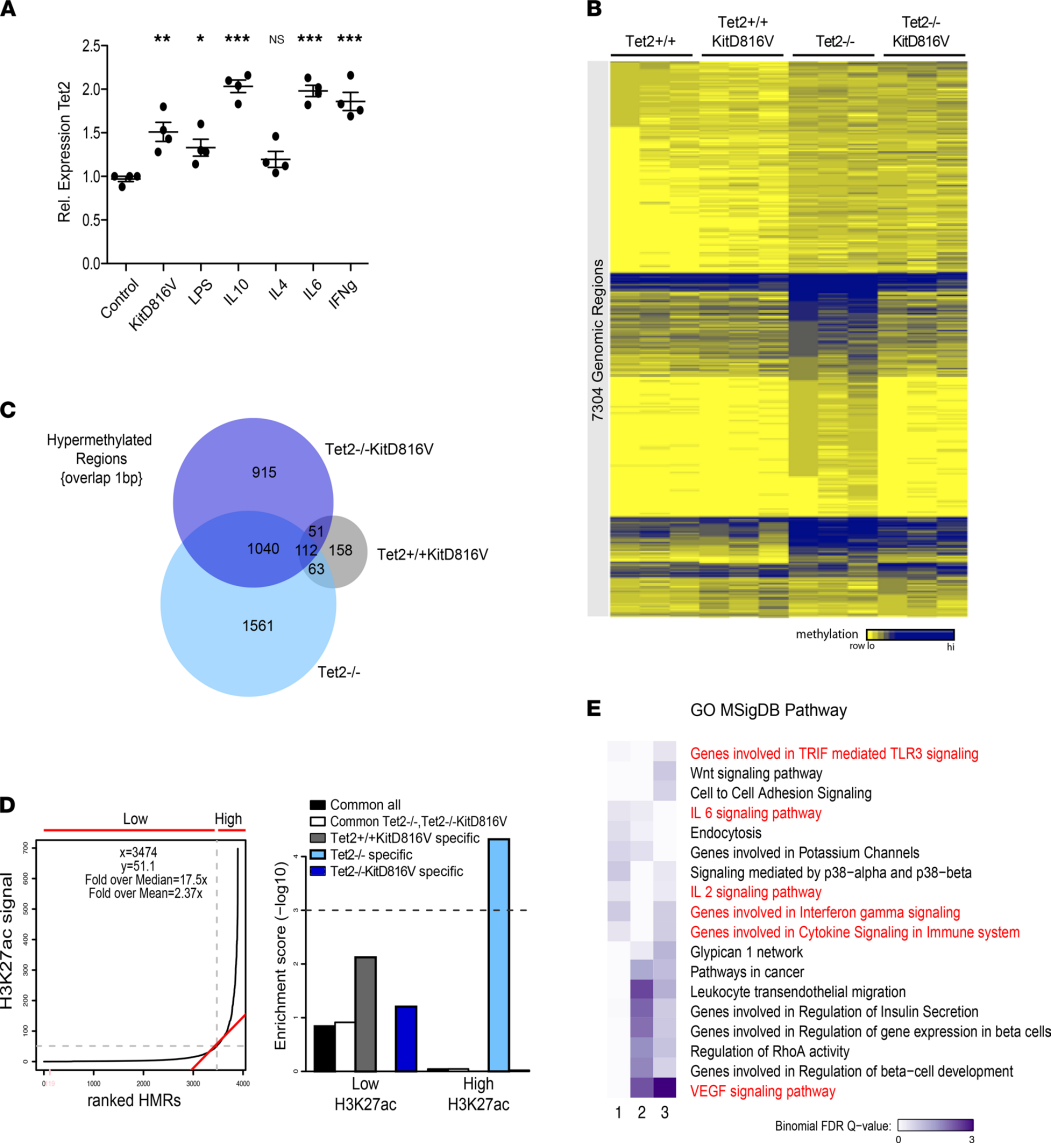
KITD816V selects for a specific methylation signature associated with immune response genes in TET2-deficient mast cells. (**A**) *Tet2* expression relative to *Hprt1* in primary unstimulated *Tet2*^+/+^ (control) and *Tet2*^+/+^*KITD816V* mast cells or *Tet2*^+/+^ cells stimulated with 10 ng/mL IL4, IL6, or IL10 or 100 ng/mL IFNγ for 4 hours (*n* = 4; mean ± SD; the means of all groups were significantly different by 1-way ANOVA; *P < 0.05, **P < 0.01, ***P < 0.001 for Dunnett’s multiple-comparison test, each treatment versus control). (**B**) Heatmap of differentially methylated regions (DMRs) determined by eRRBS analysis of biological replicates of each cell population. The first lane of each sample is the sum of the 2 independent replicates shown in other 2 lanes, and it was used for clustering. (**C**) Venn diagram showing overlap (1 bp) of hypermethylated regions (HMR) in *Tet2*^+/+^ versus *Tet2*^+/+^*KITD816V*, *Tet2*^–/–^, or *Tet2*^–/–^*KITD816V* mast cells and consistent between 2 independent biological replicates. (**D**) Histogram (left) shows DMR ranking according to H3K27ac ChIP-Seq signal. Dotted lines crossed by red slope indicate the threshold for high versus low signal subgroups. Histogram (right) shows the enrichment of DMRs associated to each genotype in either the high or low H3K27ac subgroup. Dotted line shows threshold, above which enrichment is considered statistically significant. (**E**) Heatmap showing results of gene ontology analysis for genes associated to HMR in *Tet2*^+/+^ versus *Tet2*^+/+^ *KITD816V* (lane 1), *Tet2*^–/–^ (lane 2), or *Tet2*^–/–^ *KITD816V* (lane 3) mast cells. Immune-related pathways are highlighted in red.

To determine whether *Tet2* is induced downstream of immune stimulation in primary mast cells, using this same model, we stimulated these mast cell cultures for 4 hours with different immune agonists. We observed a significant increase in endogenous *Tet2* expression in stimulated versus nonstimulated cells, and *Tet2^+/+^KITD816V* mast cells showed constitutively higher *Tet2* expression across all conditions (Figure 1A and Supplemental Figure 1A; supplemental material available online with this article; https://doi.org/10.1172/jci.insight.154191DS1).

To pursue the molecular role of TET2 in immune function, we next compared methylation signature of *Tet2*^+/+^, *Tet2*^–/–^, *Tet2*^+/+^*KITD816V*, and *Tet2*^–/–^*KITD816V* primary BM-derived mast cell cultures (referred to hereafter as *Tet2*^+/+^, *Tet2*^–/–^, *Tet2*^+/+^*KITD816V*, and *Tet2*^–/–^*KITD816V* cells). Analysis of 58,000 400 bp tiles by enhanced reduced representation bisulfite sequencing (eRRBS) identified 7304 genomic regions that were differentially methylated by at least 10% (differentially methylated regions [DMRs]) in a cross-comparison of all samples (Figure 1B). Globally, KITD816V alone had little impact on the methylation signature of mast cells; however, expression of KITD816V in *Tet2*^–/–^ cells selected for a distinct methylation signature that was both qualitatively and quantitatively different from that of *Tet2*^–/–^ cells on several levels. First, focussing on hypermethylated regions (HMR) that have been associated with TET2 loss of function, we identified 2776 HMR in *Tet2*^–/–^ cells and 2118 HMR in *Tet2*^–/–^*KITD816V* cells, of which only 46% were overlapping. Only 384 HMR were associated to KITD816V activation alone (Figure 1C). Next, using published ChIP-Seq data from WT mast cells (28), we observed a significant enrichment of HMR specific to *Tet2*^–/–^ cells at regions of high H3K27ac — also known as “super-enhancers” and often associated with lineage-determining genes (29) — and not for HMR common to *Tet2*^–/–^ and *Tet2*^–/–^*KITD816V* or specific to *Tet2*^–/–^*KITD816V* (Figure 1D). HMR associated to *Tet2*^–/–^*KITD816V* cells were significantly enriched in Erythroblast Transformation Specific factor (ETS-factor) binding motifs (Supplemental Figure 1, B and C). However, binding of the ETS-factor protein PU.1, previously identified as a TET2-interacting protein in myeloid cells (30), to DNA was similar in *Tet2*^+/+^*KITD816V* and *Tet2*^–/–^*KITD816V* (*R* = 0.97), and differences in binding did not correlate with HMR (Supplemental Figure 1D). Finally, gene-region association and ontology analysis of HMR associated to each genotype using GREAT (31) showed an increased enrichment in immune-related terms for genes associated to *Tet2*^–/–^*KITD816V* HMR compared with genes associated to *Tet2*^–/–^ HMR (Figure 1E).

Together, these data suggest that endogenous TET2 intervenes downstream of KITD816V activation, and in the absence of TET2, constitutive KITD816V activation selects for a distinct methylation profile at immune genes in primary mast cells.

### HMR and non-HMR immune gene loci are primed in Tet2^–/–^KITD816V cells.

**Figure 2:**
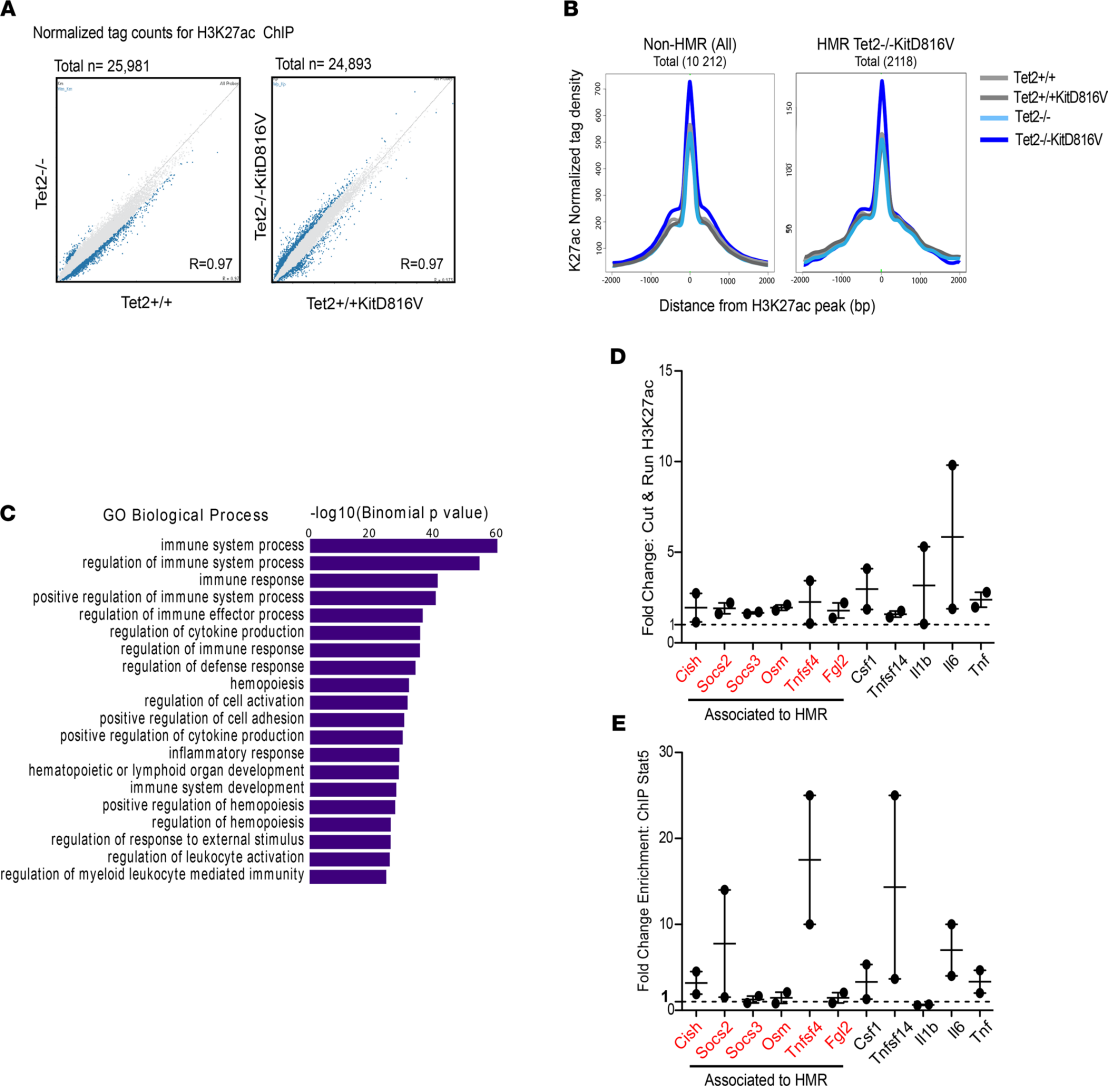
Immune response genes are enriched for H3K27ac in *Tet2^–/–^KITD816V* mast cells and at HMR. (**A**) Binary comparison of ChIP-Seq data by density scatterplot of log_2_ mean H3K27ac counts reads. Samples being compared are labeled on the *x* and *y* axes. Data are based on mean ChIP-Seq peaks of *n* = 2 replicates per sample. (**B**) Aggregation plots showing H3K27ac normalized mean tag density in each sample at non-HMR and HMR identified in *Tet2^–/–^KITD816V* cells. (**C**) Histogram showing results of gene ontology analysis for genes associated to differentially regulated H3K27ac signals in *Tet2*^+/+^*KITD816V* versus *Tet2*^–/–^*KITD816V* mast cells. (**D**) Fold change in signal at immune gene loci from Cut&Run using H3K27ac-specific antibodies in *Tet2*^–/–^*KITD816V* compared with *Tet2*^+/+^*KITD816V* mast cells. Each point is derived from 2 independent mast cell cultures for each genotype that were pooled at the time of assay. Graph show the combined results from 2 independent experiments (mean ± SEM). Immune genes also associated to HMR in *Tet2*^–/–^*KITD816V* are highlighted in red. (**E**) Fold enrichment of quantitative PCR signal in STAT5 ChIP compared with ChIP using a nonspecific IgG antibody with primers directed against STAT5 binding sites at specific gene loci. Each point is derived from 2 independent mast cell cultures for each genotype that were pooled at the time of assay. Graph shows the combined results from 2 independent experiments (mean ± SEM).

To better characterize the chromatin status at HMR in TET2-deficient cells, we performed ChIP-Seq for H3K27ac that has been associated with regulatory regions in active or open chromatin. In contrast to TET2 deficiency alone (*Tet2*^–/–^ versus *Tet2*^+/+^), *Tet2*^–/–^*KITD816V* cells showed a greater number of loci enriched for H3K27ac compared with *Tet2*^+/+^*KITD816V* cells (Figure 2A). Upon direct comparison, both HMR and non-HMR regions were enriched for H3K27ac signal in *Tet2*^–/–^*KITD816V* cells (Figure 2B). Similar to our analysis of HMR associated to *Tet2*^–/–^*KITD816V*, gene-region association and ontology analysis showed enrichment for immune terms associated to H3K27ac enriched regions in these same cells (Figure 2C). We validated this result using Cut&Run technique and show enrichment for H3K27ac at both HMR- and non-HMR–associated immune genes (Figure 2D).

Like PU.1, the transcription factor STAT5 has also been reported to interact with TET2 to regulate gene expression at DNA (32). Both KIT/SCF and KITD816V activate STAT5 as part of a downstream signaling cascade to regulate immune gene transcription in the nucleus (33, 34). Therefore, we asked whether STAT5 activation or binding to DNA at HMR- and non-HMR–associated immune genes was differentially regulated in *Tet2*^–/–^*KITD816V* cells. Basal levels of activated phospho-STAT5 in *Tet2*^–/–^*KITD816V* were higher in the steady state and in response to acute stimulation by SCF of the endogenous receptor (Supplemental Figure 2, A and B). Consistent with this, STAT5 signal measured by ChIP-PCR was also enriched at both HMR and non-HMR in *Tet2*^–/–^*KITD816V* cells (Figure 2E).

Together, both HMR and non-HMR immune gene loci are primed by both H3K27ac and STAT5 binding downstream of constitutive KITD816V signaling in *Tet2*^–/–^*KITD816V* cells.

### Tet2^–/–^KITD816V mast cells have an activated immune gene signature in the steady state.

**Figure 3:**
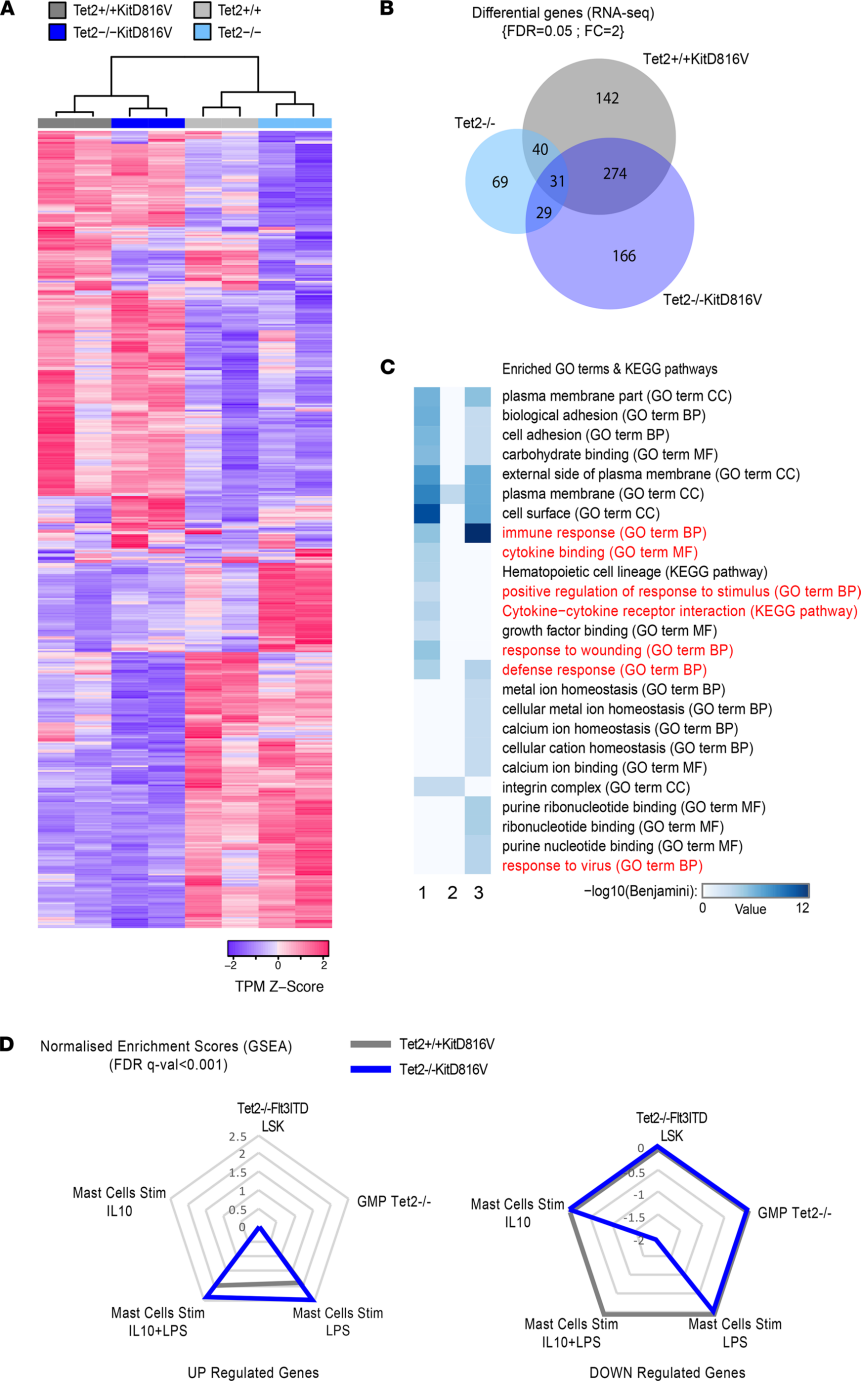
Mast cell activation genes are upregulated in *Tet2*^+/+^*KITD816V* and *Tet2*^–/–^*KITD816V* cells, but a subset of these genes is repressed in *Tet2*^–/–^*KITD816V* cells. (**A**) Heatmap of normalized RNA-Seq read counts used as signals for gene expression. The top 500 genes with highest variances among conditions were filtered in, and normalized counts were finally standardized as *Z* score. Samples and genes were clustered using Spearman and Pearson correlation, respectively. (**B**) Venn diagram showing the number of genes differentially expressed between *Tet2*^+/+^ mast cells versus *Tet2*^+/+^*KITD816V*, *Tet2*^–/–^, or *Tet2*^–/–^*KITD816V* cells as determined by RNA-Seq and consistent between 2 independent biological replicates for each cell population. (**C**) Heatmap showing results of gene ontology analysis for genes differentially expressed in *Tet2*^+/+^ versus *Tet2*^+/+^*KITD816V* (lane 1), *Tet2*^–/–^ (lane 2), or *Tet2*^–/–^*KITD816V* (lane 3) mast cells. Immune-associated pathways are highlighted in red. (**D**) Radar plots of normalized enrichment scores (NES) from gene set enrichment analysis comparing genes downregulated (lower plot) or upregulated (upper plot) in *Tet2*^+/+^ versus *Tet2*^+/+^ *KITD816V* (gray line) or *Tet2*^–/–^*KITD816V* (blue line) to genes enriched in indicated cell types compared with WT mast cells.

To investigate the impact of these epigenetic changes on gene regulation, we compared global gene expression across all 4 primary mast cell populations. In contrast to genome-wide methylation analysis, analysis of genes whose expression had the highest variance among RNA-Seq samples segregated samples according to *KITD816V* expression, as opposed to *Tet2* (Figure 3A). Relatively few significant changes (FDR 5%, absolute log_2_ fold change [log_2_FC] > 1) in the gene expression signature of *Tet2*^–/–^ compared with *Tet2*^+/+^ cells were measured compared with gene expression changes upon activation of KITD816V, both common and specific to *Tet2*^+/+^*KITD816V* and *Tet2*^–/–^*KITD816V* (Figure 3B and Supplemental Table 1).

Gene ontology analysis shows that genes that were up- and downregulated in *Tet2*^+/+^*KITD816V*, and *Tet2*^–/–^*KITD816V* compared with TET2^+/+^ cells were significantly enriched in immune-related terms. The terms associated to *Tet2*^+/+^*KITD816V* and *Tet2*^–/–^*KITD816V* were overlapping but not identical (Figure 3C). To better understand this difference, we compared up- and downregulated genes in *Tet2*^+/+^*KITD816V* and *Tet2*^–/–^*KITD816V* cells with gene signatures from immunostimulated mast cells and other TET2-deficient cell types from the public database using gene set enrichment analysis (GSEA). These analyses show a higher correlation between genes upregulated in *Tet2*^–/–^*KITD816V* cells and a unique anticorrelation for genes downregulated in *Tet2*^–/–^*KITD816V* cells and LPS- or LPS + IL-10–activated mast cells (Figure 3D). We interpret this to mean that genes that are upregulated in mast cells stimulated with LPS or LPS + IL10 are also upregulated downstream of KITD816V and to a greater extent in *Tet2*^–/–^*KITD816V* cells. On the other hand, genes that are normally high in LPS- or LPS + IL10–activated mast cells are downregulated specifically in *Tet2*^–/–^*KITD816V*V cells.

**Figure 4:**
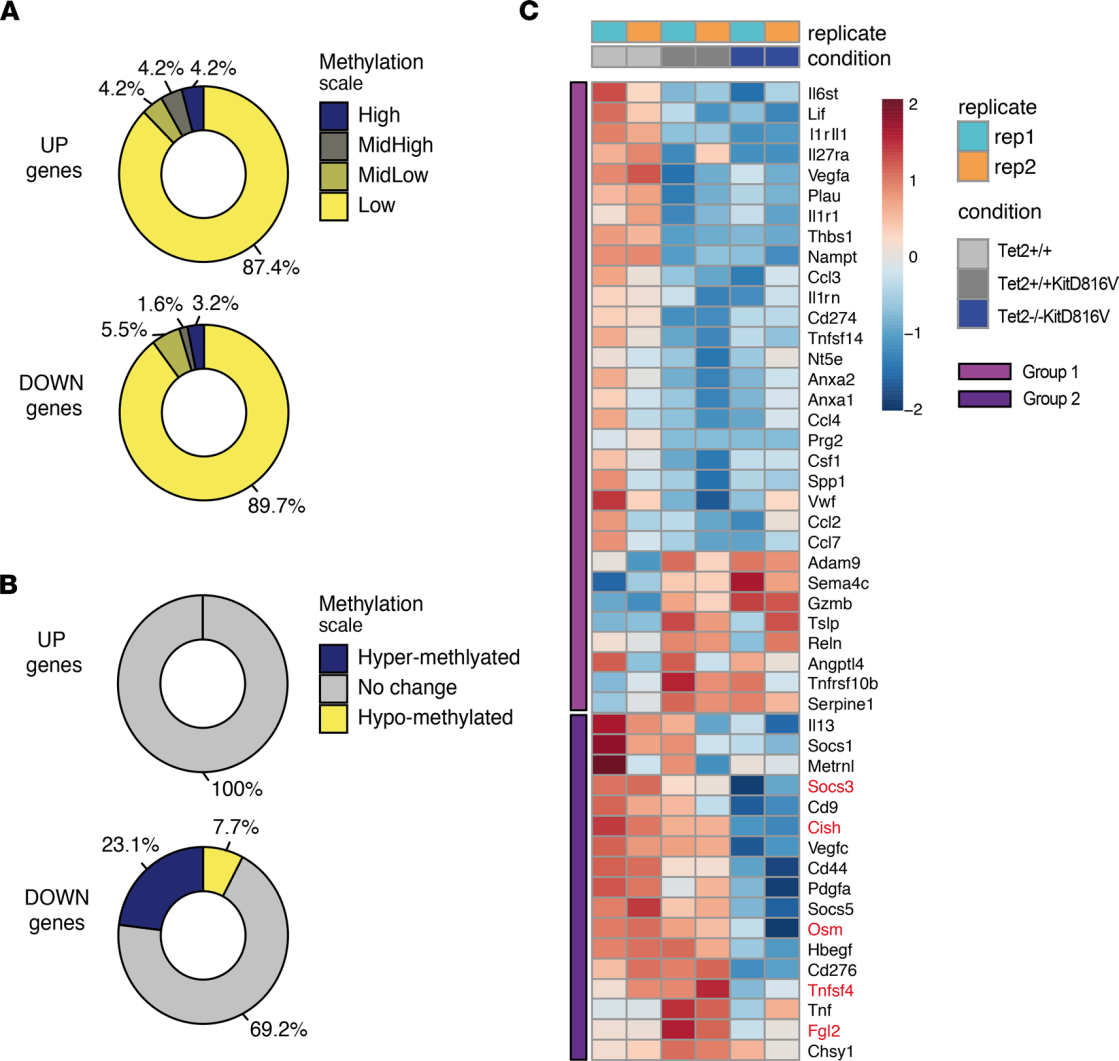
Cross-comparison of methylation and RNA-Seq data. (**A**) Differentially expressed genes (FDR 5%, absolute [log_2_FC] > 1) between *Tet2*^+/+^*KITD816V* and *Tet2*^–/–^*KITD816V* conditions were crossed with methylomics data from the same conditions. Methylation bin signals located at genes TSS (up to 1 kb upstream and downstream TSS) were filtered in and averaged per gene promoter. Methylation values were then categorized as 4 quartiles (Low [0,0.25], MidLow [0.25,0.5], MidHigh [0.5,0.75], High [0.75,1]). Bracketed numbers refer to quartile range. (**B**) As in **A**, but methylation values were categorized as 20% up (hypermethylated) or down (hypomethlated) between *Tet2*^+/+^*KITD816V* and *Tet2*^–/–^*KITD816V* conditions. Genes absent from either of the data sets were discarded. (**C**) Heatmap of scaled gene expression measured by RNA-Seq for 2 groups of mast cell activation genes described in main text. Genes associated to HMR are highlighted in red.

When we cross-compared our methylation and RNA-Seq data, there was no strict correlation between high- or low-promoter methylation and up- or downregulated genes in *Tet2*^+/+^*KITD816V* versus *Tet2*^–/–^*KITD816V* cells (Figure 4A). This is perhaps not surprising, considering that additional factors play a role in gene regulation beyond DNA methylation. Nonetheless, more downregulated genes were associated to promoter regions that were hypermethylated (i.e., HMR) in *Tet2*^–/–^*KITD816V* compared with *Tet2*^+/+^*KITD816V* cells (Figure 4B), consistent with the hypothesis that increased DNA methylation compacts chromatin to attenuate gene expression. That being said, we still do not see a strict correlation between DNA hypermethylation and downregulated gene expression changes. To explain this, we compared genes regulated downstream of KITD816V and genes regulated downstream of LPS and IL10 in mast cells with genes associated to HMR. Only 8% (44 of 531) of genes regulated downstream of KITD816V were associated to HMR, and 1% (6 genes) were common downstream of KITD816V, LPS activation, and HMR (Supplemental Figure 3). We hypothesize that the influence on gene regulation of many of the HMR might only be revealed under circumstances of specific stimulation.

To further investigate the role of TET2 mutation in the immune phenotype of *KITD816V*^+^ mast cells, we curated a list of 48 “mast cell activation” genes identified in human mast cells and detected across our murine samples (35). Thirty-one (65%, Group 1) of these genes were consistently regulated in *Tet2*^+/+^*KITD816V* and *Tet2*^–/–^*KITD816V* cells; however, 17 (35%, Group 2) of these genes were repressed in *Tet2*^–/–^*KITD816V* cells (Figure 4C). Interestingly, 5 of these repressed genes were associated to both HMR and regions of high H3K27ac (Figure 4C [highlighted in red] and Supplemental Figure 4).

Collectively, these data support a model in which both *Tet2*^+/+^*KITD816V* and *Tet2*^–/–^*KITD816V* cells share a gene signature with other activated mast cells. However, a subset of mast cell activation genes are constitutively repressed in *Tet2*^–/–^*KITD816V* cells, some of which are associated to HMR, suggesting that Tet2 may be directly involved in this regulation.

High variance and the rare number of fresh biopsies from patients with mastocytosis, TET2 mutation, and sufficient mast cell burden in the BM biopsies precluded statistical analysis of RNA-Seq on mast cells sorted from mastocytosis patients with KITD816V and TET2 mutation. Nonetheless, of the top 100 differentially regulated genes, 77% are repressed in TET2-mutated cells, as well as Group 2 mast cell activation genes detected in both murine and human mast cell samples (Supplemental Figure 5).

### Tet2^–/–^KITD816V mast cells are chronically active but refractory to acute immune stimulation.

To test the functionality of these activated mast cell signatures, we compared both chronic and acute mast cell activation responses in *Tet2*^+/+^*KITD816V* and *Tet2*^–/–^*KITD816V* cells.

**Figure 5:**
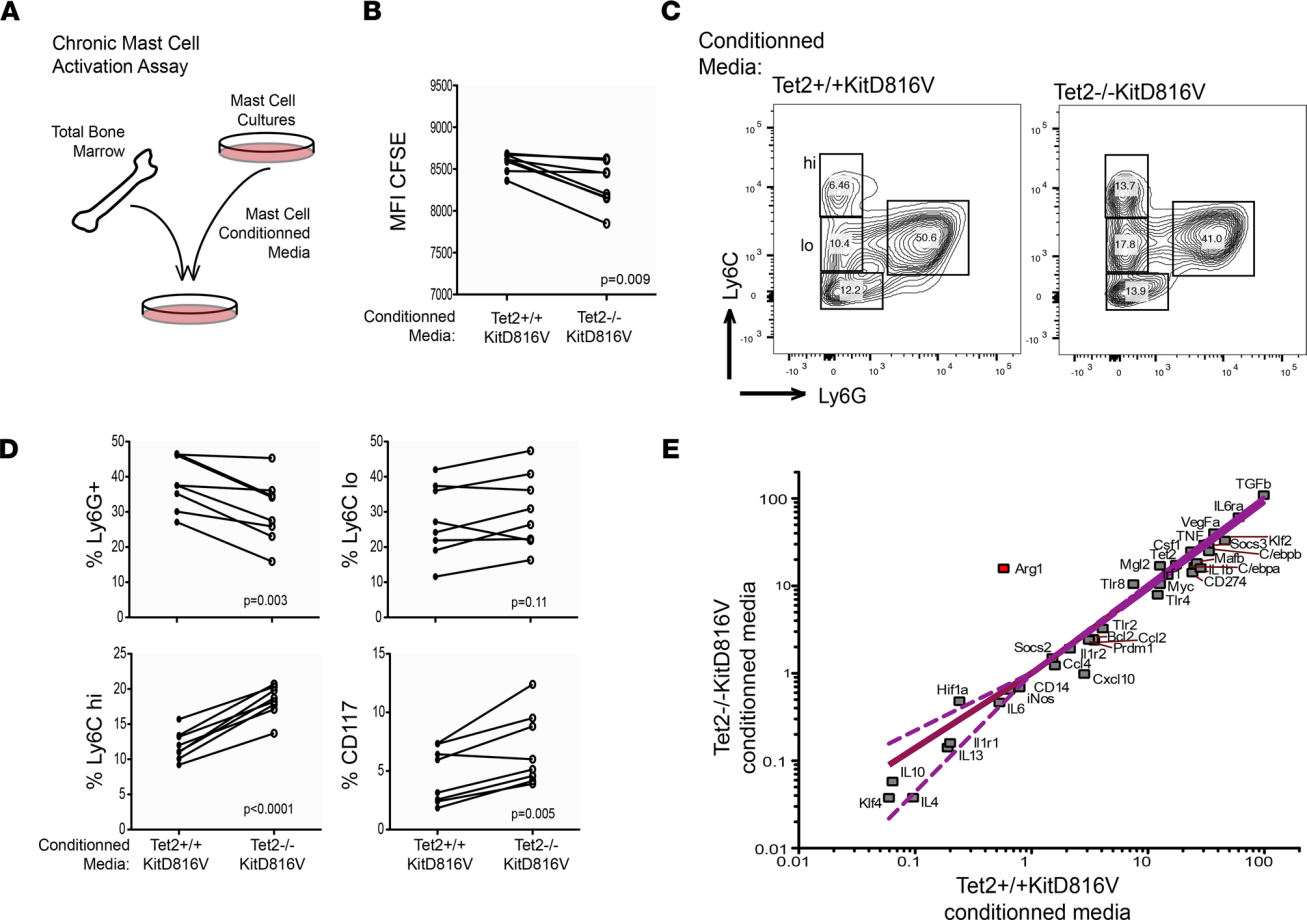
*Tet2*^–/–^*KITD816V* mast cells are chronically activated to a greater extent than *Tet2*^+/+^*KITD816V*. (**A**) Schematic showing chronic mast cell activation assay. (**B**) Quantification of mean fluorescent intensity (MFI) of Cell Trace reagent (CFSE), gated on live BM cells after 6 days of culture in conditioned media derived from either *Tet2*^+/+^*KITD816V* or *Tet2*^–/–^*KITD816V* mast cell cultures. *P* value for paired, 2-tailed *t* test is shown. (**C**) FACS analysis of BM at 3 days culture in conditioned media derived from either *Tet2*^+/+^*KITD816V* or *Tet2*^–/–^*KITD816V* mast cell cultures. Panels show representative profiles gated on live, single-cell, CD11b^+^ lymphocyte populations. (**D**) Graphs show quantification of **C** for a total of 8 paired analyses: *n* = 4 different starting BM preparations cultured with either *Tet2*^+/+^*KITD816V* or *Tet2*^–/–^*KITD816V* mast cell conditioned media, *n* = 2 each. Results shown were reproduced 3 times in independent experiments. *P* values for paired, 2-tailed *t* test are associated to each plot. (**E**) Quantification of immune genes by qPCR, relative to *Hprt1*, in total BM cultured with mast cell conditioned media for 24 hours. Each square shows mean expression for *n* = 4. Linear regression with 95% CI is shown. Slope is 0.81 ± 0.045 and shows a significant deviation from zero; *P* < 0.0001.

When activated, mast cells degranulate and/or release different inflammatory mediators into their environment based on the nature of the immune agonist encountered (36, 37). Chronic mast cell activation can also occur, often diagnosed by the abnormal clusters of “spindly” mast cells in BM biopsies of patients with mastocytosis. These neoplastic mast cells can contribute to high levels of circulating mast cell mediators detected in the blood and producing symptoms linked to chronic inflammation (12). Therefore, to further test for chronic mast cell activation, we cultured BM cells in the presence of *Tet2*^+/+^*KITD816V* or *Tet2*^–/–^*KITD816V* conditioned media (Figure 5A). Conditioned media from *Tet2*^–/–^*KITD816V* cells showed a greater proinflammatory influence on normal BM hematopoiesis. Compared with *Tet2*^+/+^*KITD816V*, conditioned media from *Tet2*^–/–^*KITD816V* cultures provoked increased CD117^+^ progenitor cell proliferation (Figure 5B), an increased proportion of Ly6C^+^ cells in the CD11b^+^ myeloid cell fraction, and significantly (Figure 5, C and D), and higher *Arg1* expression in total BM that was reproducible over multiple biological replicates from different mice (Figure 5E). Interestingly, high *Arg1* expression has been associated with myelodysplastic syndromes (MDS) and chronic myelomonocytic leukaemia (CMML) in humans, and both of these diseases are commonly associated with AHN in patients with systemic mastocytosis (38–40).

**Figure 6:**
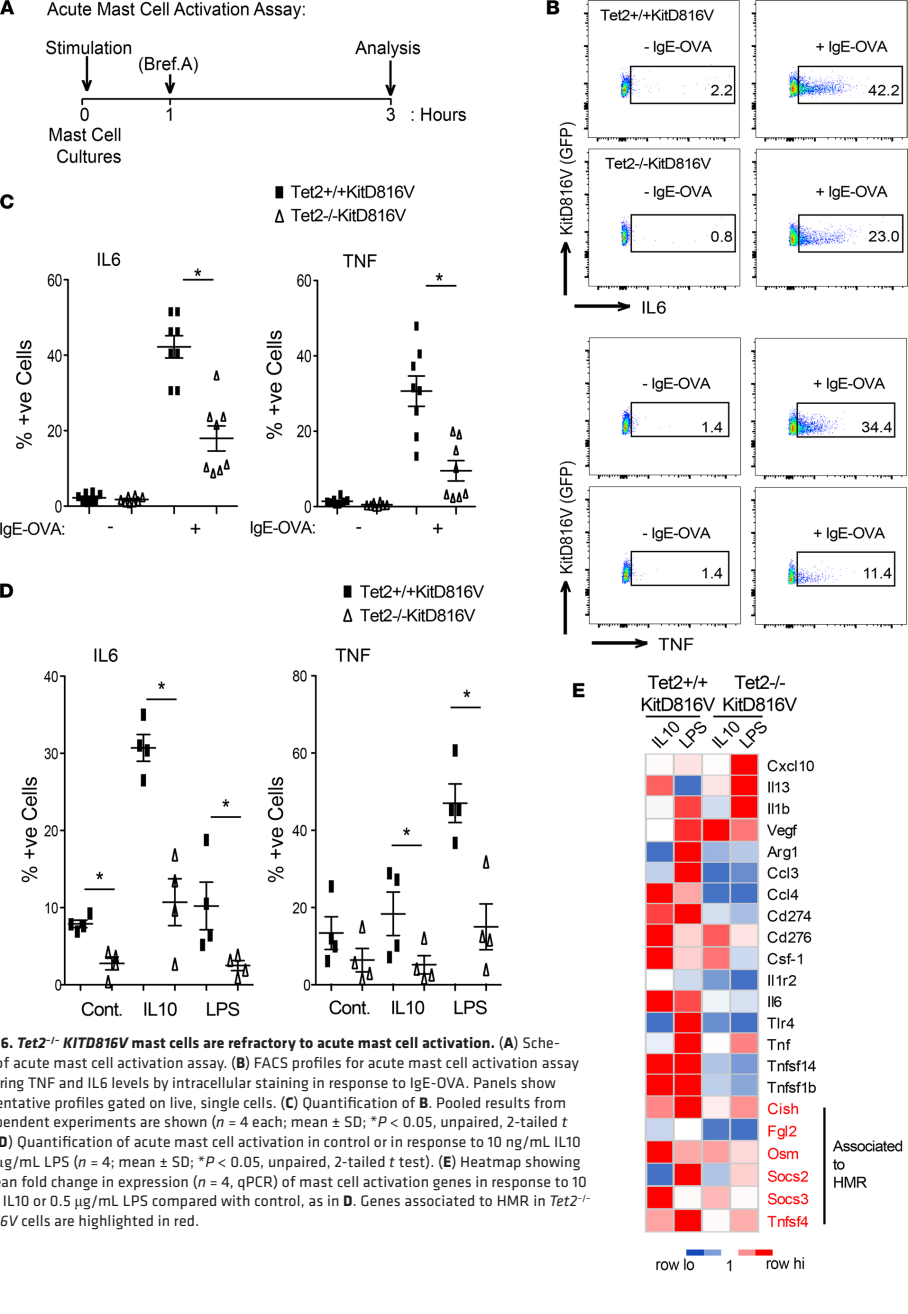
*Tet2*^–/–^*KITD816V* mast cells are refractory to acute mast cell activation. (**A**) Schematic of acute mast cell activation assay. (**B**) FACS profiles for acute mast cell activation assay measuring TNF and IL6 levels by intracellular staining in response to IgE-OVA. Panels show representative profiles gated on live, single cells. (**C**) Quantification of **B**. Pooled results from 2 independent experiments are shown (*n* = 4 each; mean ± SD; *P < 0.05, unpaired, 2-tailed *t* test). (**D**) Quantification of acute mast cell activation in control or in response to 10 ng/mL IL10 or 0.5 μg/mL LPS (*n* = 4; mean ± SD; *P < 0.05, unpaired, 2-tailed *t* test). (**E**) Heatmap showing the mean fold change in expression (*n* = 4, qPCR) of mast cell activation genes in response to 10 ng/mL IL10 or 0.5 μg/mL LPS compared with control, as in **D**. Genes associated to HMR in *Tet2*^–/–^ *KITD816V* cells are highlighted in red.

By contrast, when we performed acute mast cell activation assays (Figure 6A), *Tet2*^–/–^*KITD816V* cells were significantly less responsive compared with *Tet2*^+/+^*KITD816V* cells. Both IL6 and TNF induction were significantly lower in *Tet2*^–/–^*KITD816V* compared with *Tet2*^+/+^*KITD816V* cells in response to classical IgE-mediated crosslinking assays (Figure 6, B and C). We also performed these same assays in response to IL10 and LPS, given the results of our GSEA and the overlap between genes activated downstream of these stimuli and HMR, independent of KITD816V, and we obtained similar results (Figure 6D). Direct analysis of immune response genes revealed that, while highly induced genes such as *Il1b* and *Cxcl10* are significantly higher in *Tet2*^–/–^*KITD816V* mast cells, the majority of genes tested were refractory to stimulation in *Tet2*^–/–^*KITD816V* compared with *Tet2*^+/+^*KITD816V* cells, and these included genes associated to HMR (Figure 6E and Supplemental Figure 6).

Taken together, under steady conditions, *Tet2*^–/–^*KITD816V* cells show higher levels of chronic activation but are refractory to acute stimulation.

### TET2 can directly regulate HMR-associated immune genes, and vitamin C rescues mast cell activation.

Finally, we tested for a direct role of TET2 in the repression of immune genes associated to HMR in *Tet2*^–/–^*KITD816V* cells that might promote chronic activation and render *Tet2*^–/–^*KITD816V* cells refractory or tolerant to acute stimulation downstream of other agonists.

**Figure 7:**
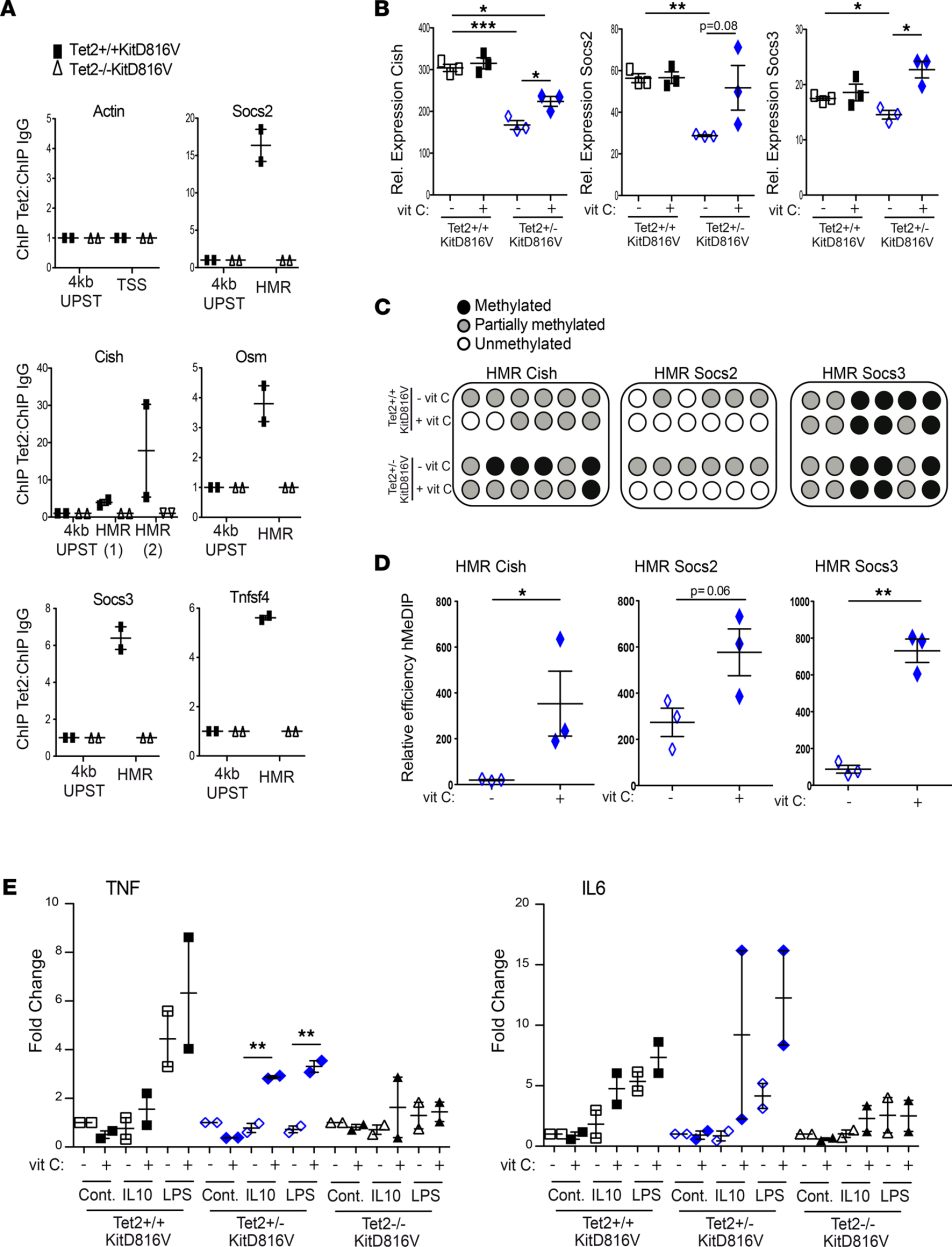
TET2 is required to directly regulate mast cell activation genes and rescue acute mast cell activation. (**A**) ChIP-qPCR analyses showing enrichment in signal in TET2 ChIP over ChIP using a nonspecific IgG antibody at HMR and at regions 4 kb upstream the HMR in *Tet2*^+/+^*KITD816V* (gray) and *Tet2*^–/–^*KITD816V* cells (blue; negative control for TET2 antibody). Each point is derived from 3 independent mast cell cultures for each genotype that were pooled at the time of assay. Graphs show the combined results from 2 independent experiments (mean ± SEM). (**B**) Expression levels of genes relative to *Hprt1* in untreated *Tet2*^+/+^*KITD816V* and *Tet2* ^+/–^*KITD816V* mast cells or after 24 hours of treatment with 250 μM vitamin C (*n* = 3; mean ± SD; the means of all groups were significantly different by 1-way ANOVA; *P < 0.05, **P < 0.01, ***P < 0.001 for Dunnett’s multiple-comparison test, each treatment versus control, or unpaired, 2-tailed *t* test to compare *Tet2* ^+/–^*KITD816V* with or without vitamin C. Only significant differences are shown in the figure). (**C**) Representative methylation profile of HMR associated regions in *Tet2*^+/+^ *KITD816V* and *Tet2* ^+/–^*KITD816V* mast cells obtained by performing bisulfite sequencing. Samples were untreated or treated for 24 hours with 250 μM vitamin C. Black circles represent methylated CpG dinucleotide, while gray and white circles indicate partially methylated or unmethylated CpGs, respectively (mean methylation of *n* = 3). (**D**) Enrichment of 5hmC at HMR associated loci in *Tet2* ^+/–^*KITD816V* mast cells with or without vitamin C treatment (250 μM for 24 hours) evaluated by using hydroxy-methyl DNA immunoprecipitation (hMeDIP) (*n* = 3; mean ± SD; *P < 0.05, **P < 0.01 unpaired, 2-tailed *t* test). (**E**) Quantification analysis for acute mast cell activation assay measuring TNF and IL6 levels by intracellular staining. Cells were pretreated for 24 hours with 250 μM vitamin C, prior to acute mast cell activation assay using 10 ng/mL IL10 or 0.5 μg/mL LPS as in Figure 5A. Fold change is relative to control for each cell population. Each point is derived from 3 independent mast cell cultures for each genotype that were pooled at the time of assay. Graph show the combined results from 2 independent experiments (mean ± SD; **P < 0.01 unpaired, 2-tailed *t* test).

We first confirmed that TET2 associated with DNA directly targeted the HMR-associated genes that we had consistently validated in our assays. Indeed, by ChIP, we detect endogenous TET2 protein in *Tet2*^+/+^*KITD816V* mast cells at regions that correspond to HMR in *Tet2*^–/–^*KITD816V* cells (Figure 7A).

Next, because ectopic expression of *Tet*2 in primary mast cells was toxic in our hands, we used vitamin C to rescue TET2 function in *Tet2* heterozygous (*Tet2* ^+/–^) mast cells. Vitamin C has been shown to act as a cofactor to boost activity of the remaining TET2 allele to rescue the haploinsufficient phenotype of other *Tet2* ^+/–^ hematopoietic cells (41–43), and *Tet2* ^+/–^*KITD816V* mast cells have an intermediate proliferation and survival phenotype compared with *Tet2*^+/+^*KITD816V* and *Tet2*^–/–^*KITD816V* cells (8).

Rescue for gene expression changes downstream of TET2 in KITD816V-expressing cells was strongest and most consistent for the 3 *Socs* genes associated to HMR (Figure 7B and Supplemental Figure 7A). Immune genes not associated to HMR had a variable and nonspecific response to vitamin C treatment. *SOCS* genes are of particular interest in the context of *KITD816V* expression, since SOCS proteins function as part of a retro-feedback loop to resolve tyrosine kinase receptor signaling. Suppression of *Socs* genes could, therefore, provide a molecular explanation for the increase in chronic signaling downstream of KITD816V and protracted STAT5 activation that we measure in *Tet2*^–/–^ mast cells. We further tested for rescue of DNA demethylation and hydroxymethylation at *Socs* genes using vitamin C in *Tet2* ^+/–^*KITD816V* compared with *Tet2*^+/+^*KITD816V* cells. Consistent with increased TET2 activity, vitamin C treatment resulted in either decreased DNA methylation or increased DNA hydroxymethylation, as well as rescue of gene expression in all cases (Figure 7, C and D).

We further tested the ability of vitamin C to more broadly rescue the diminished acute activation phenotype in *Tet2*^–/–^*KITD816V* cells. A caveat to these experiments is that vitamin C can act as a cofactor to enzymes other than TET2, including TET3, in the immune system, reducing the specificity of these “rescue” experiments, as evidenced by the effects of vitamin C in TET2-nullizygous cells in some of our assays (44). Nonetheless, vitamin C treatment was able to rescue for TNF production that was specific to cells expressing TET2; however, vitamin C increased IL6 production nonspecifically in activated mast cells (Figure 7E).

To further support a direct role for TET2 in regulating mast cell activation, we performed the inverse experiment and inhibited endogenous TET proteins using Teti76 (45). We show that treating cells with Teti76 for 24 hours prior to activation suppressed TNF and IL6 induction upon acute stimulation of mast cells (Supplemental Figure 7B). Again, we do see some activity with this inhibitor in *Tet2*^–/–^*KITD816V* cells, suggesting that, like vitamin C, Teti76 could have additional effects via TET1/3 inhibition or other off targets.

## Discussion

Previous studies that have described the epigenetic signatures associated with TET2 loss of function in immature hematopoietic cells, alone or in combination with other driver mutations in the context of a leukemic landscape, where TET2 has been associated with the regulation of lineage specification and differentiation genes (6, 7, 27). Here, we show that expression of the *KITD816V* allele in mature primary mast cells deficient for TET2 selects for a specific epigenetic signature associated with immune response genes and the pathological inflammation associated with mastocytosis.

From a clinical perspective, this is important because we show that, compared with mast cells transformed by *KITD816V* alone, TET2-deficient *KITD816V* transformed mast cells secrete factors that have a greater influence on the differentiation of myelosuppressor cells with high *Arg1* expression from total BM. This has clear implications for the prognosis of patients carrying TET2 mutations where AHN, often present in patients with aggressive forms of systemic mastocytosis, could progress from crosstalk between TET2/KITD816V mutated mast cells and myeloid progenitor cells in the BM. Overall, our data show that activation of TET2 could provide a new therapeutic option to improve prognosis of the > 30% of patients with aggressive mastocytosis associated with these 2 mutations.

Mechanistically, we show that, in the presence of constitutive KITD816V signaling, TET2 is required to induce a subset of immune genes to prevent a broader immune tolerance in these chronically activated cells. Unlike previous studies that profiled DNA methylation in TET2-deficient mast cells, we show that this signature is profoundly changed upon activation of the KITD816V driver mutation associated with mastocytosis. HMR scored in *Tet2*^–/–^*KITD816V* cells overlapped with only about half of those scored in *Tet2*^–/–^ cells, and almost as many new HMR were revealed. Given the strong clonal selection for cells expressing the *KITD816V* allele in TET2-deficient cells (8), it is equally possible that HMR specifically detected in *Tet2*^–/–^*KITD816V* cells are novel or underrepresented in original *Tet2*^–/–^ populations. Our data show that TET2 was not required at HMR to open chromatin. Indeed, H3K27ac was constitutively higher at both HMR and non-HMR in *Tet2*^–/–^*KITD816V* cells, reflecting the increase in chronic activation in these cells. STAT5 has also been reported to interact with TET2 (32), and here we show that STAT5 binding at DNA downstream of KITD816V was both independent of TET2 and insufficient for full gene activation at these immune targets. We focussed on this mechanism of regulation by TET2 at *Socs* genes since there exists a clear mechanistic link between KITD816V, STATs, and *SOCS* genes in mast cell biology. *SOCS* genes are broadly engaged downstream of receptor pathway activation in the hematopoietic system. For example, where somatic deletion of either *Socs1* or *Socs3* in mice is lethal (46–48), myeloid-specific deletion of these factors potentiates inflammation as a result of deregulated macrophage polarization in response to proinflammatory triggers (49). *Cish* is induced by TCR stimulation in CD8^+^ T cells and genetic deletion of *Cish* in these cells enhances their expansion, immune reactivity, and cytokine repertoire (50). Interestingly, if the molecular link between *TET2* and *CISH* gene regulation is also true in T cells, this could provide a molecular explanation for preferential expansion ex vivo upon TET2 mutation in CAR T cells (51, 52) and provides additional rationale for targeting TET2 and SOCS for immune therapies. However, our efforts to recapitulate the *Tet2*^–/–^*KITD816V* phenotype in single *Socs* gene–KO models were inconclusive, and this may be because the broad changes to the expression and epigenetic landscape that we measured in *Tet2*^–/–^*KITD816V* compared with *Tet2*^+/+^*KITD816V* cells cannot be recapitulated through the bias of a single downstream target.

We have used a model of somatic mutation of *Tet2* for our studies, since in mastocytosis, clonal mutation of *TET2* occurs during early hematopoiesis and *KITD816V* mutation is secondary (8, 53). Because *TET2* expression is high in stem cells, one model is that TET2 regulates DNA methylation in early progenitor cells, presumably with lasting effects in differentiated progeny where TET2 levels are generally lower. However, we show that endogenous TET2 is induced in activated mast cells and can bind to immune gene loci to regulate DNA methylation in these cells. Moreover, we can rescue the mast cell activation in *Tet2* ^+/–^*KITD816V* cells, at least to some extent, with vitamin C, showing that we can intervene on this phenotype in mature cells. Taken together, it will be important to pursue specific TET2 inhibitors that can be used to block TET2 function transiently during inflammation to fully discern the roles of TET2 during inflammation and hematopoiesis.

Finally, while a role for TET2 in the inflammation response in myeloid cells has now been accepted in the field, here we report a tolerant phenotype associated to the immune function of TET2-deficient cells. Trained immunity and tolerance are 2 facets of innate immune memory. Though the molecular mechanisms of trained immunity and tolerance are not well elucidated, epigenetic signatures have been described that correlate with training and immune memory in myelomonocytic and stem cells (54–56). While these signature can be stimulus specific, trained immunity can have broader effects upon second stimulation with different agonists. This appears to be the case for *Tet2*^–/–^*KITD816V* cells that are chronically activated downstream of KITD816V but refractory to acute stimulation in response to other disparate agonists. Globally immune genes were epigenetically primed and upregulated in *Tet2*^–/–^*KITD816V* cells, likely driven by the chronicity of inflammatory signals (57); however, repressed genes associated with both HMR and TET2 loss of function provided a common denominator in the steady state and in response to agonist that was associated to tolerance.

On this note, although *Tet2*^–/–^*KITD816V* mast cells are less reactive overall, we observed higher levels of activated expression in *Tet2*^–/–^*KITD816V* mast cells of several proinflammatory genes, *Cxcl10*, *Il13*, and *Il1b*, and strong suppression of *Arg1*, usually associated to alternative macrophage activation, in response to stimulation. This is similar to a study showing that, in the tumor niche, TET2-deficient TAMs select for a proinflammatory signature and prevents the alternative/immunosuppressive phenotype of macrophages that supports tumor growth (16). However, classical and alternative mast cell activation is not well defined, so it’s difficult to reconcile a role for TET2 in immune tolerance and the differentiation of specific mast cell subsets (58). It will be important to resolve this distinction and how it applies to mast cells and macrophages since, on the one hand, rescue of TET2 function would be beneficial to resolve chronic inflammation, while on the other hand, transient inhibition of TET2 might serve in resisting immune suppressive signals in innate immune cells for cancer therapy.

Overall, it will be interesting to study the role of HMR in controlling tolerance and whether dynamic regulation of this epigenetic signature by TET2 during inflammation plays a role in trained immunity in other contexts. Future studies will also be aimed at resolving whether TET2 hydroxymethylation of DNA is required to recruit additional cofactors to immune targets for full transcription activation, and if so, whether they are gene or stimulus specific.

## Methods

Next-generation sequencing data sets are deposited as Super series GSE122686 (https://www.ncbi.nlm.nih.gov/geo/query/acc.cgi?acc=GSE122686).

For a list of main reagents, oligonucleotide sequences (primers for quantitative PCR [qPCR]), software, and algorithms used, please refer to Supplemental Table 2.

### Cell culture

Primary mast cells were derived from BM of mice as described in ref. 8. Total BM from TET2-deficient or WT littermates were differentiated in the presence of IL3 for 10–15 days and were then infected with lentivirus carrying the *KITD816V* allele and GFP (IRES) (high-titer viral particles produced by SFR Biosciences, UMS3444/CNRS, US8/Inserm, ENS de Lyon, UCBL, Lyons, France). Infected populations were the either sorted by FACS on FACSAria using DIVA (Becton-Dickinson) software for direct lysis and RNA or DNA isolation, further expanded as KITD816V^+^ or KITD816V^–^ populations, or kept as mixed populations for FACS analysis of GFP^+^ and GFP^–^ subpopulations under experimental conditions. Cells were cultured for 24 hours in the presence of L-Ascorbic acid (vitamin C, MilliporeSigma, A4544) or Teti76 (synthesized in-house, according methods provided in ref. 59) where indicated in figure legends.

### BM and mast cell conditioned media assay

Conditioned media was obtained from mast cell cultures seeded at a density of 500,000 cells/mL in fresh mast cell media and cultured for 72 hours. BM was flushed from femurs and tibias of 8- to 10-week-old WT C57BL/6J mice, washed 1 time in PBS, and then directly resuspended in mast cell conditioned media for up to 6 days. For proliferation assays, RBCs were lysed using ACK, and leukocytes were then labeled with Cell Trace Violet (Invitrogen) according to manufacturer instructions, prior to resuspension in mast cell conditioned media. Both adherent and nonadherent cells were collected for analysis at time points indicated and stained with the following fluorochrome-conjugated antibodies: CD117 (ACK2, eBioscience), CD11b (M1/70, eBioscience), Ly6G (1A8, BD Pharmingen), and Ly6C (AL-21, BD Biosciences). Data shown are representative of *n* = 6 independent BM replicates with conditioned media from *n* = 4 independently derived mast cell cultures.

### RNA-Seq (mouse)

Primary murine mast cells were culture as described for 30 days, and RNA was isolated from sorted GFP^+^ and GFP^–^ cell fractions using the RNeasyPlus kit (Qiagen) for sequencing. Eight indexed libraries were prepared using the Illumina TrueSeq protocol following ribodepletion, and 100 single-end reads were run on a HiSeq2500 from Illumina (iGE3 Genomics Platform, University of Geneva, Geneva, Switzerland).

### RNA-Seq (human)

Fresh or frozen whole BM biopsy material from patients was stained with the following antibody cocktail: anti–human CD3-ECD (UCHT1, BD Biosciences), anti–human CD14-Alexa 647 (M5E2, BioLegend), anti–human CD25-PE (BC96, BD Biosciences), and anti–human FcɛR1a-FITC (AER37[CRA-1], BioLegend). Cells were sorted on an FACSAria using DIVA (Becton-Dickinson) software. Sorted cell populations were directly lysed, and RNA was isolated using the RNeasyPlus Micro KIT (Qiagen) and sent for sequencing. Libraries were prepared using the Illumina TrueSeq protocol: Smarter+Nextera, 6-indexed libraries multiplexed 1 lane of an Illumuna HiSeq2500, sequencing for 50 single-end reads (iGE3 Genomics Platform, University of Geneva, Geneva, Switzerland). Primary murine mast cells were cultured as described for 30 days, and RNA was isolated from sorted GFP^+^ and GFP^–^ cell fractions using the RNeasy Plus kit (Qiagen) for sequencing. Eight indexed libraries were prepared using the Illumina TrueSeq protocol following ribodepletion, and 100 single-end reads were run on a HiSeq2500 from Illumina (iGE3 Genomics Platform, University of Geneva, Geneva, Switzerland).

### Gene expression analysis

RNA was isolated using the RNeasy Plus KIT (Qiagen) and reverse transcribed using oligo dT_12–18_ primers together with Superscript III (Invitrogen). qPCR was performed using Sso-Advanced Universal SYBR Green Supermix (Bio-Rad) on a CFX96 Touch Real-Time PCR Detection System (Bio-Rad). qPCR primer pairs are listed in Supplemental Methods.

### DNA methylation analysis

Bisulfite sequencing was performed using the using EZ DNA Methylation-Gold KIT (Zymo Research). For hydroxymethyl DNA immunoprecipitation (hMeDIP), genomic DNA was isolated using the DNeasy kit (Qiagen). gDNA (500 ng for each sample) was sonicated with a Bioruptor Sonicator (Diagenode) for 45 cycles (30 seconds on /30 seconds off, low energy mode) at 4°C. Hydroxymethylcytosine immunoprecipitation of sheared samples was performed using EpiQuick hMeDIP KIT (Epigentek). Socs2, Socs3, and Cish HMR IP were quantified by qPCR.

### Phospho-FACS

Primary mast cells were cultured in OptiMEM/0.5% FCS for 3 hours prior to stimulation with 250 ng/mL SCF (Peprotech). Cells were collected at indicated time points after stimulation and assayed for STAT5 phosphorylation using phospho-specific antibody, Alexa Fluor 647 mouse phospho-STAT5 (pY694, clone 47, Becton Dickinson) directly coupled to fluorochromes, using the PerFix EXPOSE (Beckman Coulter) staining reagents and protocol for whole blood samples. Cells were analyzed on a BD LSRFortessa X-20 or LSRII and analyzed using DIVA (Becton-Dickinson) and FlowJo software.

### Intracellular cytokine staining

In total, 2.5 × 10^5^ cells/mL were stimulated with 250 ng/mL SCF and either IgE anti-DNP 0.5 μg/mL and DNP-OVA 0.2 μg/mL, 10 ng/mL recombinant murine IL10 (Peprotech) or 0.5 μg/mL LPS (MilliporeSigma) for 3 hours. Brefeldin A 10 μg/mL was added in the last 2 hours of stimulation to inhibit protein exocytosis. Cells were fixed Cytofix/Cytoperm (BD Pharmingen) and stained with anti–TNF-α/PE-Cy7 (MP6-XT22, BioLegend) or –IL6/PE (MP5-20F3, BioLegend) on ice for 20 minutes. Cells were washed, resuspended in 500 μL of Perm/Wash, and analyzed by flow cytometry using the BD LSRII or Fortessa system.

### ChIP

Primary mast cells cultures were fixed for ChIP as described in ref. 60. Each batch of 10 million cells was lysed and processed using reagents and according to the protocol provided by the ChIP-IT High Sensitivity (HS) KIT (Active Motif). Two million cell equivalents were used for each ChIP. The following antibodies were used: H3K4me1 (Abcam, ab8895), H3K27ac (Abcam, ab4729), PU.1 (Santa Cruz Biotechnology, sc-352), ERG-1/2/3 (Santa Cruz Biotechnology, sc-354), antibody against total STAT5 (Santa Cruz Biotechnology, sc-835), and TET2 (Proteintech, 21207-I-AP). ChIP samples were quantified using the Qubit Fluorometric quantification System (Thermo Fisher Scientific) and using a DNA high-sensitivity DNA kit on a Bioanalyzer (Agilent). Target enrichment was analyzed by qPCR or processed for NGS.

### Cut&Run

Protocol was adapted from ref. 61 using 5 × 10^5^ primary mast cells for each tested condition and using the following antibodies: H3K27ac (Abcam, ab4729) and STAT5 (Santa Cruz Biotechnology, sc-835). Cut&Run products were quantified by qPCR.

### RRBS

Primary murine mast cells were infected and cultured as described for 30 days, and genomic DNA was isolated from sorted GFP^+^ cell fractions using the DNeasy kit (Qiagen) for RRBS. RRBS libraries were prepared as described previously (62) with minor modifications. Genomic DNA (50–200 ng) was digested for 5 hours with MspI; end-repaired; A tailed, where a single adenine base was added to form an overhang via an A-tailing reaction; and ligated with T4 DNA ligase (Fermentas) to methylated Illumina adaptors. In total, 150–400 bp fragments were gel purified and bisulfite treated (EpiTect Bisulfite kit, Qiagen), and RRBS libraries were amplified by 15 cycles of PCR with PfUTurbo Cx hotstar DNA polymerase (Agilent) and indexed PE Illumina primers. The libraries were paired-end sequenced (2 × 75 bp) on a HiSeq-2000 to an average of 30 million pairs of reads per sample.

### ChIP-Seq

Libraries were made using the Multiplex Library Preparation KIT (Diagenode). Nine cycles of PCR were used for all libraries. Each ChIP-Seq library was sequenced for single-end reads on 1 lane of Illumina HiSeq 4000. Biological replicates for ChIP-Seq were performed on independently derived primary mast cell cultures from 2 different mice for each sample and both sequenced. Comparison of ChIP-Seq and gene expression data revealed no substantive differences between biological replicates (*R* ≥ 0.97).

### RNA-Seq analysis

Sequencing quality control was done with FastQC v.0.10.1. Reads were mapped with the TopHat v2.0.11 software to the UCSC mouse (mm10) or human (GRCh38/hg38) reference genome, with, on average, 20 million aligned reads/sample for mice and 25 million for humans. Biological quality control and summarization were done with RSeQC v2.4, PicardTools v1.92, and SamTools v0.1.18. Read coverages were determined to be uniform, and the percentage of mRNA bases was, on average, 64%. The percentage of ribosomal bases was less than 5%. The count data were prepared with HTSeq v.0.6.1p1 (htseq-count, default parameters). The normalization and differential expression analysis was performed with the R/Bioconductor edgeR package v.3.4.2. The raw count data were filtered. We filtered out very lowly expressed genes, keeping genes that are expressed at a reasonable level. Since the group size average is 2, we kept genes that achieved 10 counts in at least 2 samples: genes 23,420–11,821 = 11,599 expression values for each sample, which came to about 49% (mouse); genes 23,710–14,298 = 9,412 expression values for each sample, which came to about 40% (human). The filtered data were normalized by the library size, and differentially expressed genes were estimated with the negative binomial general model statistics.

### RRBS analysis

Raw reads were cleaned with Trim Galore (v0.2.1) and aligned to the mm10 genome with BSMAP (v2.74). We identified DMRs with the eDMR algorithm from the methylKIT R package with the following criteria: at least 3 CpGs, a difference in methylation greater than 20%, and an adjusted *P* < 0.01. DMRs were annotated using the RefSeq mm10 transcript annotation.

### GSEA

Public data sets used included GSE48085 (WT mast cells and HPC7) GSE55385 (Mast cells stimulated with LPS/IL10), GSE57244 (GMP, TET2^–/–^Flt3ITD LSK), and GSE10246 (CMP). Reads were aligned using the mm10(GRCm38) mouse genome assembly, and a table of read counts was established for each gene using the STAR tool. Differential analysis was done using the edgeR package v.3.4.2 with the following thresholds: FDR = 0.05, fold change (FC) = 2. The GSEA tool was then used to compare differential gene sets. Radar plots were generated in Excel.

### ChIP-Seq analysis

Sequencing quality control was done with FastQC v0.11.6. Cleaning up raw sequencing reads was performed with sickle v.0.6.3 (–q 20, –l 25 parameters). Cleaned reads were mapped with the Bowtie2 V2.3.4.1 software to the mm10 (GRCm38), with, on average, 37 million, 35 million, and 38 million aligned reads/sample for PU.1, H3K27ac, and H3K4me1, respectively. SAM files where converted to BAM files and indexed using samtools V1.7. The peak calling was carried out using MACS version 2.1.1.20160309 software (–q 0.01 for PU.1 ChiP, --broad for H3K27ac and H3K4me1 ChIP). BedGraph control subtracted files were obtained using bdgcmp from the same version of MACS. Finally, bedGraph files were sorted and converted to bigwig files using bedGraphToBigWig V.4 for IGV visualization purpose. Downstream analysis of peaks was done using R V3.4.1, DiffBind R/Bioconductor package V2.6.6, ggplot2 V3.0.0, and bedtools V2.27.1. Gene region associations were assigned using GREAT.

### H3K27ac enrichment in HMR analysis

H3K27ac ChIP-Seq data were recovered from GSE48085 (bedGraph data). HMR were translated from mm10 to mm9 mouse genome assembly using the Liftover tool (UCSC). Average H3K27ac profiles were generated by extracting ChIP-Seq signal from bedGraph file around HMR’s center (±2.5 kb). Threshold for “Low” was set at 0.5. The “High” H3K27ac group was determined by identifying an inflection point of the average H3K27ac signal versus DMRs rank. The inflection point was computed by determining the diagonal line of the curve from end points and by sliding this diagonal line to find where it is tangential. Enrichment scores were calculated using the Fisher exact *t* test.

### Statistics overview

For biological assays, statistics were calculated using Prism 6 software (GraphPad Software Inc.). Unless otherwise indicated in figure legend, the significance of the differences between groups was determined by unpaired, 2-tailed *t* tests. *P < 0.05; **P < 0.01; ***P < 0.001. When comparing multiple samples with a same control (Figure 1A and Figure 7B), 1-way ANOVA followed by Dunnett’s multiple-comparison test was performed to determine significance. All replicates are biological replicates: independently derived BM mast cell populations from individual mice of indicated genotype, each infected in parallel with lentivirus carrying the KITD816V allele.

### Study approval

#### Mice.

C57BL/6J TET2^LacZ^ mice were obtained from O. Bernard and T. Mercher (4) (Institut Gustave Roussy, Villejuif, France). Animals were housed in an specific opportunist and pathogen-free–certified (SOPF-certified) facility, and all experiments were performed in compliance with the laws and protocols approved by animal ethics committees (Authorization/Certification no. D1305504, PréfectureBouches-du-Rhône, France). Both male and female mice, between the ages of 10 and 15 weeks, were grouped for analysis. Independent analysis of data derived from male and female mice did not show significant differences, with the exception of RRBS data, where XY chromosome data were excluded for DMR analysis.

#### Patient data.

Patients with mastocytosis diagnosis as defined by the WHO criteria were enrolled in a prospective national multicenter study between 2005 and 2013. KIT and TET2 mutation analysis for this cohort have been presented elsewhere (8). All patients were included in a mastocytosis pathophysiological study that started in 2003 and is sponsored by the Association For Initiative and Research on Mast cell and Mastocytosis (AFIRMM). The study was approved by the Necker Hospital ethical committee and carried out according to the Declaration of Helsinki. Each patient provided informed consent.

## Author contributions

Conceptualization was contributed by ES and PD. Methodology was contributed by ES, PD, OAB, and MW. Investigation and Validation were contributed by ES, RR, JA, SL, HG, GG, EV, and VL. Software, formal analysis, and data curation were contributed by AG, RC, JV, GB, and MW. Writing of the original draft was contributed by RR and ES. Review and editing the manuscript were contributed by RR, ES, PD, OAB, and JAN. Resources were contributed by MW, BG, GB, GG, PDS, and JAN. Supervision was contributed by ES and PD. Project administration was contributed by ES. Funding acquisition was contributed by ES, PD, OAB, and MW.

## Supplementary Material

Supplemental data

## Version 1 04/08/2022

Electronic publication
