# Supplementary material for: TET2 regulates immune tolerance in chronically activated mast cells
Source: JCI Insight. 2022 Apr 8;7(7):e154191. doi: 10.1172/jci.insight.154191 (PMC9057605; doi:10.1172/jci.insight.154191)
Supplement: Supplemental data [file jciinsight-7-154191-s241.pdf]

Supplementary Table S1

Summary list from RNA-seq analysis listing the total number of regulated genes for each comparison.

Comparison	# Significant genes (at 5% FDR)	# down regulated	# up regulated	# Significant genes (at 5% FDR + FC ≥ 2)	# down regulated (FC ≥ 2)	# up regulated (FC ≥ 2)
**1)** Tet2-/- vs Tet2-/-KitD816V	4,322	2,282	2,040	716	590	126
**2)** Tet2+/+ vs Tet2+/+KitD816V	2,724	1,530	1,194	487	426	61
**3)** Tet2+/+ vs Tet2-/-	664	402	262	169	131	38
**4)** Tet2+/+KitD816V vs Tet2-/-KitD816V	323	161	162	98	56	42

Supplementary Table S2. Main reagents

REAGENT or RESOURCE	SOURCE	IDENTIFIER
**Antibodies**		
Alexa Fluor® 647 Mouse phospho-Stat5 (pY694) Clone 47	Becton Dickinsin	562076
H3K4me1	Abcam	ab8895
H3K27ac	Abcam	ab4729
PU.1	Santa Cruz	sc-352
Stat5	Santa Cruz	sc835
Tet2	Proteintech	21207-I-AP
IL6 -PE	BioLegend	MP5-20F3
TNFa-PECy7	BioLegend	MP6-XT22
CD117	eBioscience	ACK2
CD11b	eBioscience	M1/70
Ly6C	BD Bioscience	AL-21
Ly6G	BD Bioscience	1A8
**Critical Commercial Assays**		
**ChIP-IT High Sensitivity® (HS) Kit**	Active Motif	53040
EZ DNA Methylation-Gold™ Kit	Zymo Research	D5006
EpiQuick hMeDIP Kit	Epigentek	P-1038-24
Bio-Plex Pro™ Mouse Cytokine 8-plex assay	BioRad	M60000007a

**Deposited Data:** Super series GSE122686<br><br>**deposited at** https://www.ncbi.nlm.nih.gov/geo/query/acc.cgi?acc=GSE122686		
**Experimental Models: Organisms/Strains**		
C57BL/6J Tet2^{LacZ} mice (referred to as Tet2-/-)	(*1*)	
**Oligonucleotides (Q-PCR) mRNA**		
Hprt1 F: CTCGAGATGTCATGAAGGAGATG	This paper	n/a
Hprt1 R: TTCAGTGCTTTAATGTAATCCAG	This paper	n/a
Arg1 F: CATGGGCAACCTGTGTCCTT	This paper	n/a
Arg1 R: CGATGTCTTTGGCAGATATGCA	This paper	n/a
Irf1 F: TCTTGCCCTCCTGAGTGAGT	This paper	n/a
Irf1 R: GGGACTATGCTTTGCCATGT	This paper	n/a
TNF F: TAGCCAGGAGGGAGAACAGA	This paper	n/a
TNF R: TTTTCTGGAGGGAGATGTGG	This paper	n/a
Vegfa F: CCACGACAGAAGGAGAGCAGAAGTCC	This paper	n/a
Vegfa R: CGTTACAGCAGCCTGCACAGCG	This paper	n/a
Mmp9 F: TATAGCTACCTCGAGGGCTTCC	This paper	n/a
Mmp9 R: GTGGGAGGTATAGTGGGACACA	This paper	n/a
Cxcl10 F: AAGTGCTGCCGTCATTTTCT	This paper	n/a
Cxcl10 R: CCTATGGCCCTCATTCTCAC	This paper	n/a
Mcp2 F: GCGCAGGCAGTCCCACAACA	This paper	n/a
Mcp2 R: CGGGTGAAGACTGCAGGGGC	This paper	n/a
Mcp6 F: GGGTCAGGCAAGAACCAGGGC	This paper	n/a

Mcp6 R: GGGAGGCAAGAGGGAACCGGA	This paper	n/a
Socs2 F: ATGCACTGGGTCAAAAGTCC	This paper	n/a
Socs2 R: CAAGCATGGTCAGCTTAACG	This paper	n/a
Socs3 F: CTTTTCTTTGCCACCCACGG	This paper	n/a
Socs3 R: CCGTTGACAGTCTTCCGACA	This paper	n/a
OSM F: AGGGGTCTGATGACACAAGC	This paper	n/a
OSM R: AGTGTGAGGTCACCCAGAGG	This paper	n/a
IL4 F: TCACAGCAACGAAGAACACC	This paper	n/a
IL4 R: TTGCATGATGCTCTTTAGGC	This paper	n/a
IL13 F: ATTGCATGGCCTCTGTAACC	This paper	n/a
IL13 R: TGAGTCCACAGCTGAGATGC	This paper	n/a
Bcl2 F: CCTGTGGATGACTGAGTACCTG	This paper	n/a
Bcl2 R: AACAGAGGTCGCATGCTGG	This paper	n/a
Socs1 F: CCGCTGCAGGAGCTGTGTCG	This paper	n/a
Socs1 R: GAGGGATGCGCGCCAGGTTC	This paper	n/a
Cish F: ACATCCTGGAGGGAACACAG	This paper	n/a
Cish R: CCTAGAGGCGTTGACCTCAG	This paper	n/a
Ctnnb1 F: ACGCACCGTCCTTCGTGCTG	This paper	n/a
Ctnnb1 R: AGGCGAACGGCATTCTGGGC	This paper	n/a
Tet2 F: AACCTGGCTACTGTCATTGCTCCA	This paper	n/a
Tet2 R: ATGTTCTGCTGGTCTCTGTGGGAA	This paper	n/a
Tet3 F: TCCGGATTGAGAAGGTCATC	This paper	n/a
Tet3 R: CCAGGCCAGGATCAAGATAA	This paper	n/a

**Oligonucleotides (Q-PCR) gDNA - ChIP/Cut&Run**		
Cish HMR (1) F: GCAACCTTTTCACACCGACC	This paper	n/a
Cish HMR (1)R: CCCACTAAGCCAAAGAGGGG	This paper	n/a
Cish HMR (2) F: TGAGCACACTTCCAGCTCAG	This paper	n/a
Cish HMR (2)R: GGAAGTCCCCTCATCAAGGC	This paper	n/a
Socs2 HMR F: CAGAATGGTGTGGCAAAGTCTC	This paper	n/a
Socs2 HMR R: TGACCCAATCTCTGTTCCATTGT	This paper	n/a
Socs3 HMR F: GGAGAGACAGCGGTCGTAAG	This paper	n/a
Socs3 HMR R: GCGTACTGGCCGGGTAAATA	This paper	n/a
Cish UPST F: ACAGCTGGACCAGAGAATGC	This paper	n/a
Cish UPST R: AGGCGTTGACCTCAGAGTTG	This paper	n/a
Socs2 UPST F: GCATAGTCTGCTCTCGGGAC	This paper	n/a
Socs2 UPST R: AAATTGTACCCTGCTTGGCG	This paper	n/a
Socs3 UPST F: CCGTAAGCCAGGGTACATCC	This paper	n/a
Socs3 UPST R: GTTCAAAATCCGGCTGGTGC	This paper	n/a
Csf-1 F: AGCTCTGGACAAGACACTGC	This paper	n/a
Csf-1 R: GATTTTCGGGCCTTGAGGGA	This paper	n/a
Osm F: TCGTGTCCTAGGAGGTTGGT	This paper	n/a
Osm R: CGGGGGTCCGTATCATCTTC	This paper	n/a
IL6 F: CCCCGAAGGCAAAGAGACTT	This paper	n/a
IL6 R: TGTATCTTGATCCTGGCCGC	This paper	n/a
IL1b F: ACGAGAGGGAAGGGGTGTAA	This paper	n/a

Il1b R: TTGCATCCTGGCTACTCACC	This paper	n/a
Tnfsf14 F: AGTAGGTCAATGTCTTGGCCG	This paper	n/a
Tnfsf14 R: ATCCCGGAGGTGTAGGCATT	This paper	n/a
Tnf F: CCCCGCTTACAGTTCCTCTTT	This paper	n/a
Tnf R: CAGAAAGAAGCCGTGGGTTG	This paper	n/a
Fgl2 F: CGGCAGGCATTCTATTGTGC	This paper	n/a
Fgl2 R: GCGCATTTGCATCACAGTCT	This paper	n/a
Tnfsf4 F: TGGCTGATATTTGTTGACCCC	This paper	n/a
Tnfsf4 R: TTTCTCGGAAGGCTGGTGTG	This paper	n/a
Bclx F: TTCCCCTTCCCCCACCATTA	This paper	n/a
Bclx R: CCCCAGCGAGGTGTTATCTC	This paper	n/a
**Software and Algorithms**		
FastQC		http://www.bioinformatics.babraham.ac.uk/projects/fastqc/
TopHat	(*2*)	
RSeQC	(*3*)	
Picard Tools		http://picard.sourceforge.net/
Samtools		http://samtools.sourceforge.net/

IGV		http://www.broadinstitute.org/igv/home
HTseq		http://www.huber.embl.de/users/anders/HTSeq/
edgeR	(*4*, *5*)	
Samtools		http://samtools.sourceforge.net/
GREAT	(*6*)	

1. C. Quivoron, L. Couronné, V. Della Valle, C. K. Lopez, I. Plo, O. Wagner-Ballon, M. Do Cruzeiro, F. Delhommeau, B. Arnulf, M.-H. Stern, L. Godley, P. Opolon, H. Tilly, E. Solary, Y. Duffourd, P. Dessen, H. Merle-Beral, F. Nguyen-Khac, M. Fontenay, W. Vainchenker, C. Bastard, T. Mercher, O. A. Bernard, TET2 Inactivation Results in Pleiotropic Hematopoietic Abnormalities in Mouse and Is a Recurrent Event during Human Lymphomagenesis. *Cancer Cell*. **20**, 25–38 (2011).
2. C. Trapnell, L. Pachter, S. L. Salzberg, TopHat: discovering splice junctions with RNA-Seq. *Bioinformatics*. **25**, 1105–11 (2009).
3. L. Wang, S. Wang, W. Li, RSeQC: quality control of RNA-seq experiments. *Bioinformatics*. **28**, 2184–5 (2012).
4. D. J. McCarthy, Y. Chen, G. K. Smyth, Differential expression analysis of multifactor RNA-Seq experiments with respect to biological variation. *Nucleic Acids Res.* **40**, 4288–97 (2012).
5. M. D. Robinson, D. J. McCarthy, G. K. Smyth, edgeR: a Bioconductor package for differential expression analysis of digital gene expression data. *Bioinformatics*. **26**, 139–40 (2010).
6. C. Y. McLean, D. Bristor, M. Hiller, S. L. Clarke, B. T. Schaar, C. B. Lowe, A. M. Wenger, G. Bejerano, GREAT improves functional interpretation of cis-regulatory regions. *Nat. Biotechnol.* **28**, 495–501 (2010).

Supplemental Figure S1

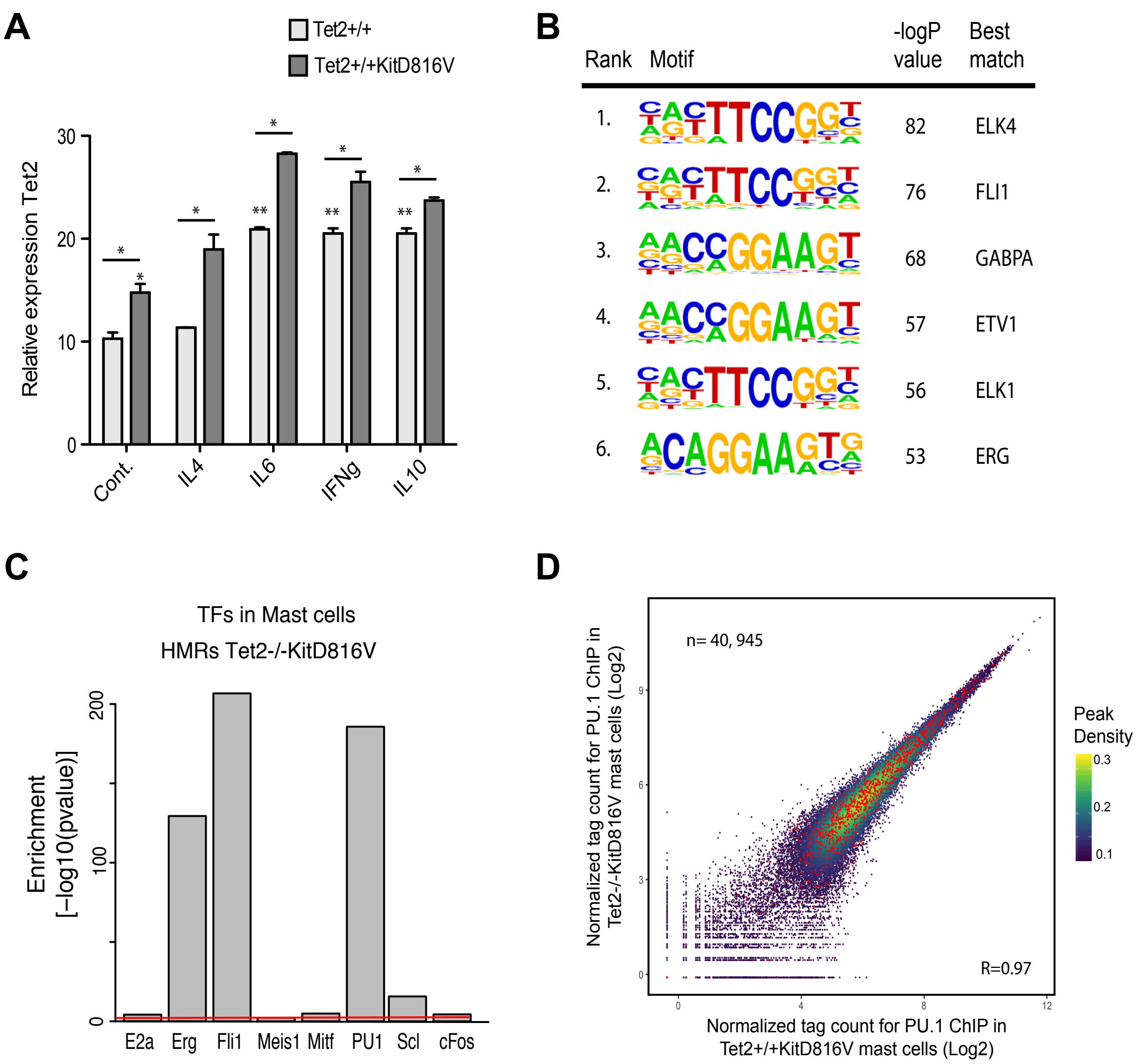

Figure S1. HMR are enriched for Ets-factor bindng motifs in Tet2-/-KitD816V cells.
(A) Tet2 expression relative to HPRT1 in primary Tet2+/+ and Tet2+/+KitD816V mast cells unstimulated (Cont.) or stimulated with 10 ng/ml IL4, IL6, IL10 or 100 ng/ml IFNg for 4 hours (n = 3; mean ± SD; * p<0.05, **p<0.01, unpaired two-tailed t test). **(B)** Top enriched sequence motifs in HMRs associate with Tet2-/-KitD816V mast cells. **(C)** Calculated enrichment for transcription factor binding at HMRs associated with Tet2-/-KitD816V in wild type mast cells. Red line indicates a threshold for significant enrichment (FDR<0.05). **(D)** Density scatterplot of log2 mean counts reads in Tet2+/+KitD816V and Tet2-/-KitD816V cells for PU.1 enriched peaks. Total number of peaks called (n) and normalized reads similarity (R) are shown. Red dots identify PU.1 peaks within 5kb of HMR in Tet2-/- KitD816V (n=800). Data are based on mean ChIP-seq peaks of the replicate samples.

Supplemental Figure S2

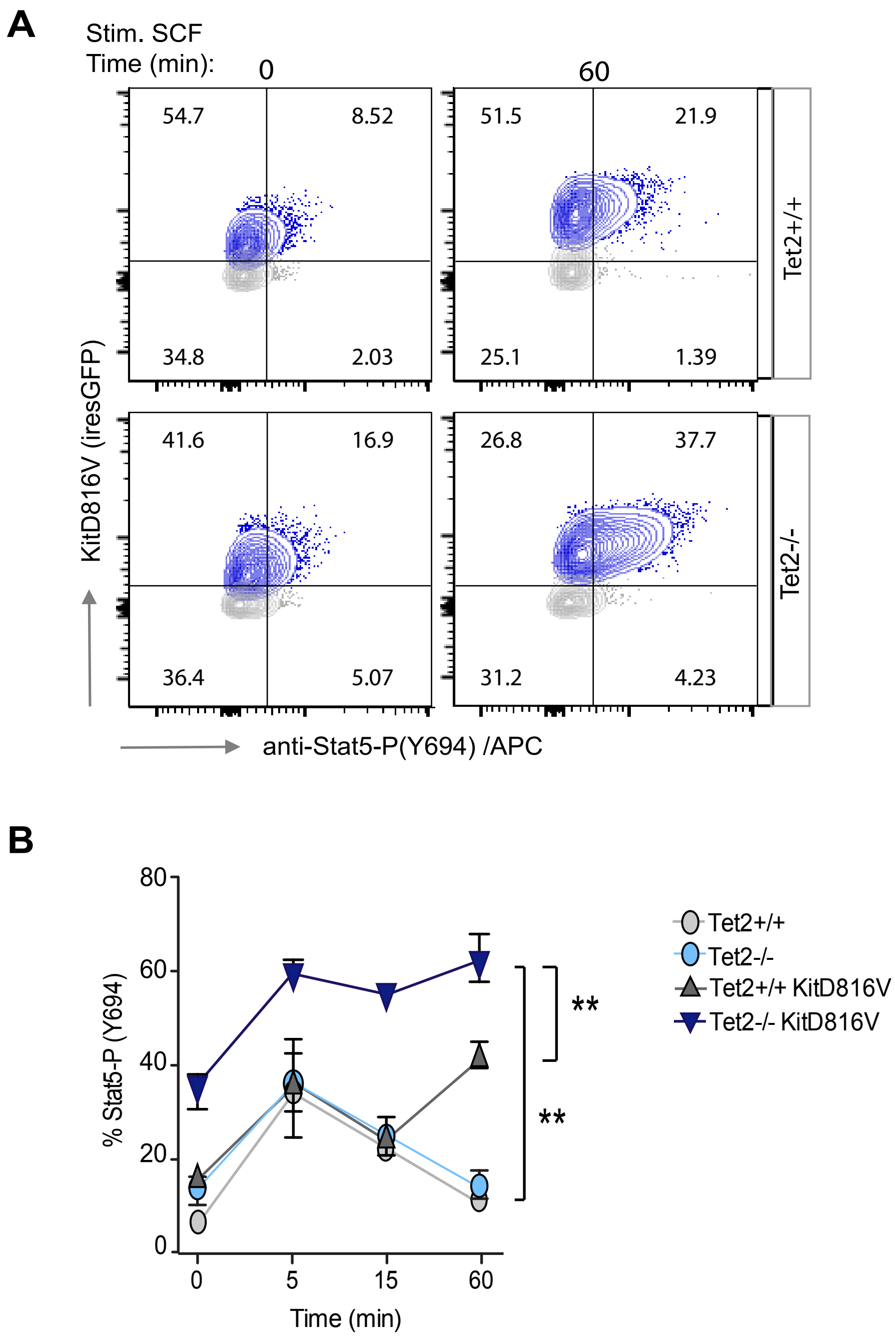

Figure S2. Increased Stat5 activation downstream of KitD816V signalling in Tet2-deficient cells. (A) Phospho FACS analysis of Stat5-P(Y694) in mast cells positive and negative for KitD816V and stimulated with 250 ng/ml SCF for indicated times. **(B)** Plot showing mean ± SEM quantification of (A) over time, n=3 biological replicates and data shown are representative of at least two indepen -dent experiments. (** $p<0.01$, unpaired two-tailed t-test).

Supplemental Figure S3

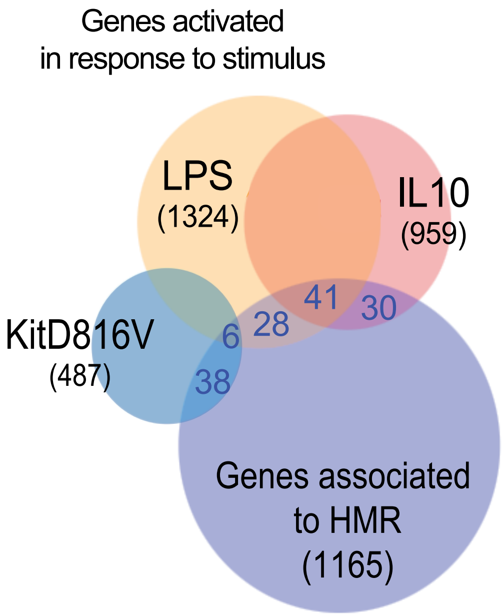

Figure S3. Venn Diagram showing the overlap of genes regulated downstream of KitD816V (Tet2+/+KitD816V vs Tet2+/+ cells, RNAseq data, Figure 3), LPS and IL10 stimulated mast cells (public Geneset GSE55385) and genes associated to hypermethylated regions (HMR) in Tet2-/-KitD816V versus Tet2+/+ cells (based on eRRBS data, Figure 1).

Supplemental Figure S4

Gene Regions Associated to HMR

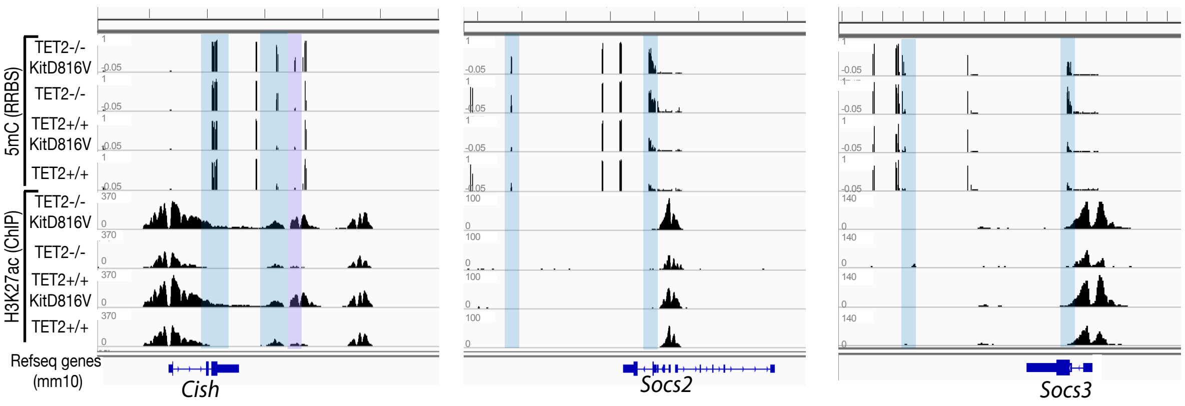

Gene Regions Not associated to HMR

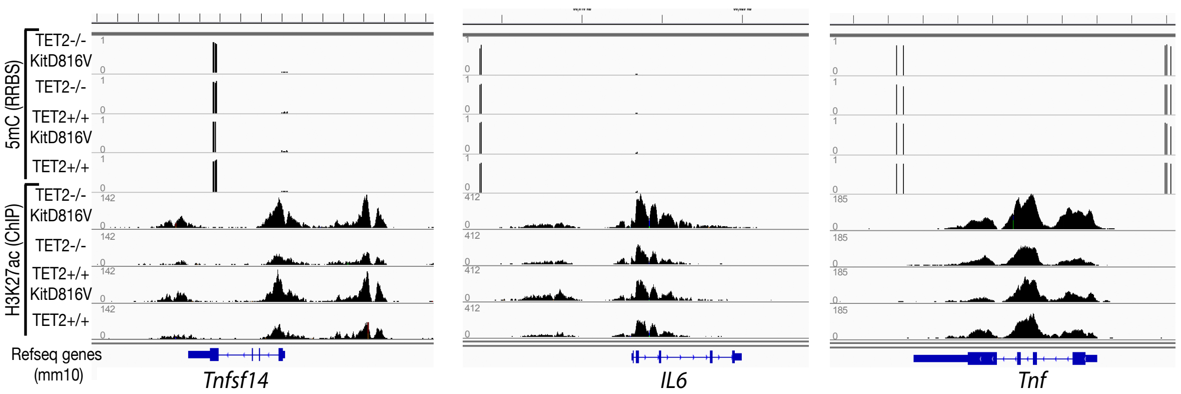

Figure S4. Genes repressed in KitD816V cells are associated to both HMR and non-HMR and regions of high H3K27ac. IGV images showing representative profiles for 5-methyl cytosine (5mC) and H3K27ac signals mapped to the mm10 mouse reference genome at immune genes associated (upper row) or not associated (lower row) to HMR inTet2-/-KitD816V cells in all four primary mast cell populations. HMR scored in Tet2-/-KitD816V cells are shaded across all samples.

Supplemental Figure S5

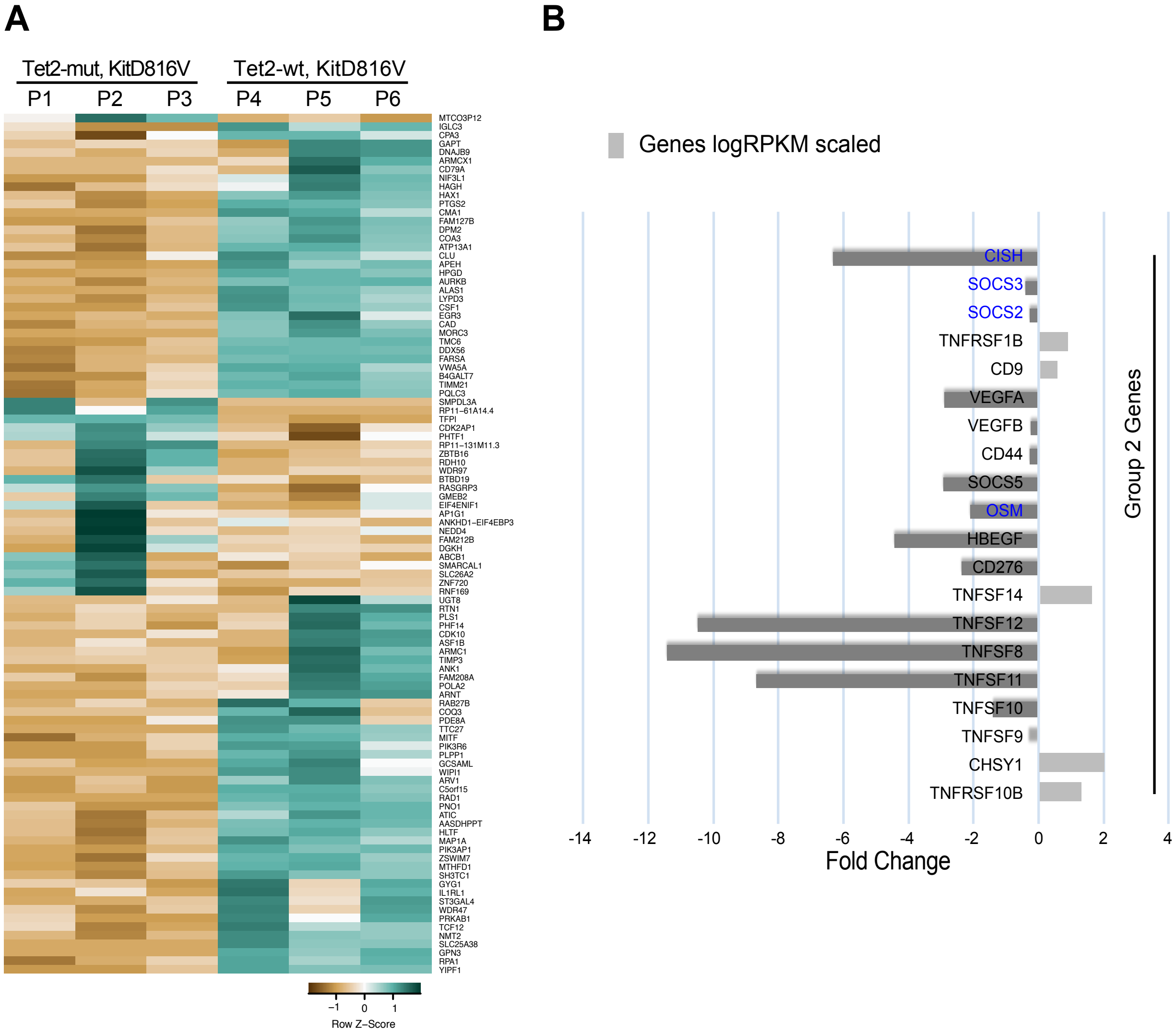

Figure S5. (A) Clustered heatmap for top one hundred genes differentially regulated in mast cells sorted from bone marrow of patients (P1-P6) with aggressive forms of systemic mastocytosis. The KitD816V mutation was detected in all patients, and at least one mutation in Tet2 was detected in samples from patients P1-P3, no Tet2 mutations were detected in samples from patients P4-P6. **(B)** Average FC between for logRPKM scaled values in P1-P3 compared to P4-P5, of mast cell activation genes from groups 2 as describe in Figure 3E (main text).

Supplemental Figure S6

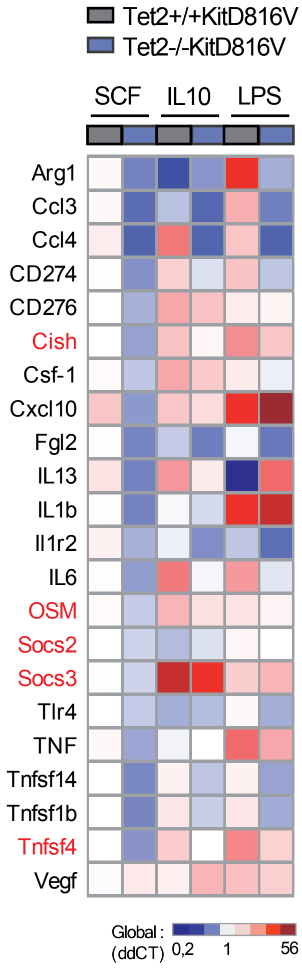

Figure S6. Heatmap of gene expression data presented in Figure 6E on a global scale, also showing baseline response of control cells to SCF alone. Mean fold change in expression (n=4, RT-QPCR) in response to 10ng/ml IL10 or 0.5ug/ml LPS. Genes associated to HMR in Tet2-/-KitD816V cells are highlighted in red.

Supplemental Figure S7

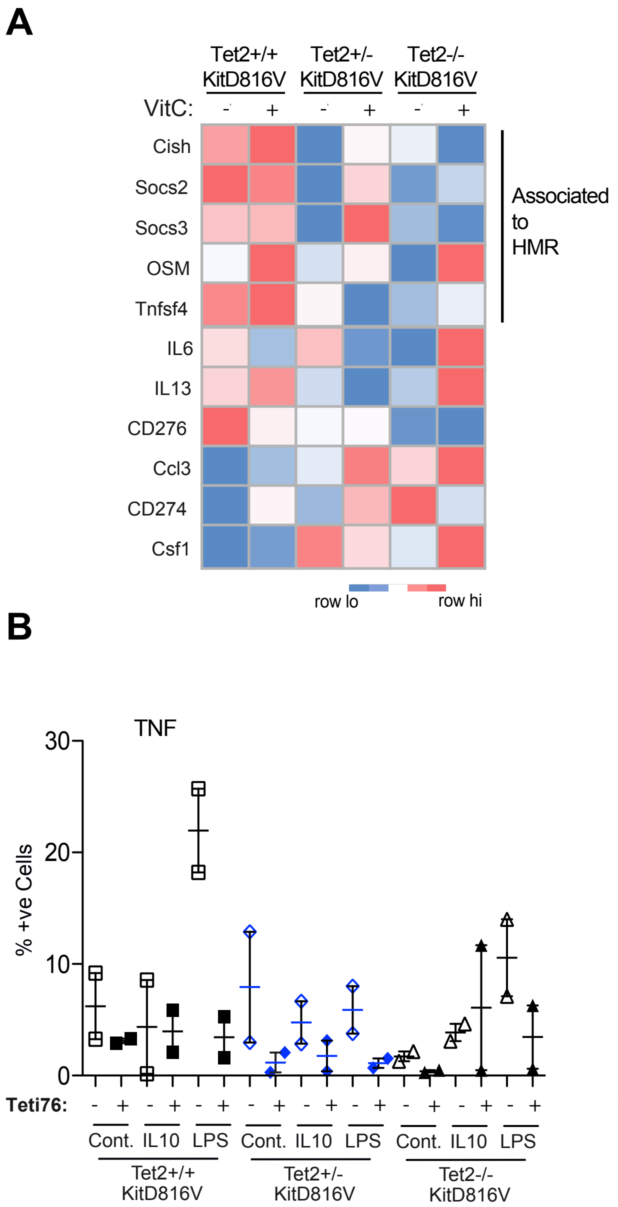

Figure S7. (A) Heatmap showing the mean expression (n=4) relative to HPRT of mast cell activation genes treated or untreated 24h with 250 µM vitamin C, quantified by RT-QPCR. **(B)** Quantification analysis for acute mast cell activation assay measuring TNF and IL6 levels by intracellular staining. Cells were pre-treated for 24h with 25 µM Teti76, prior to acute mast cell activation assay using 10ng/ml IL10 or 0.5ug/ml LPS as in Figure 5A. Each point is derived from three independent mast cell cultures for each genotype that were pooled at the time of assay. Graph shows the combined results of two independent experiments (mean ± SD).
